# The intracellular parasite Anncaliia algerae induces a massive miRNA down-regulation in human cells

**DOI:** 10.1016/j.ncrna.2023.05.003

**Published:** 2023-05-18

**Authors:** Reginald Florian Akossi, Fréderic Delbac, Hicham El Alaoui, Ivan Wawrzyniak, Eric Peyretaillade

**Affiliations:** a Laboratoire “Microorganismes: Génome et Environnement” (LMGE), UMR 6023, Université Clermont Auvergne and CNRS, F-63000, Clermont-Ferrand, France

## Abstract

Anncaliia algerae belongs to microsporidia, a group of obligate intracellular parasites related to fungi. These parasites are largely spread in water and food-webs and can infect a wide variety of hosts ranging from invertebrates to vertebrates including humans. In humans, microsporidian infections are mainly opportunistic as immunocompetent hosts can clear parasites naturally. Recent studies however have reported persistent microsporidian infections and have highlighted them as a risk factor in colon cancer. This may be a direct result of cell infection or may be an indirect effect of the infectious microenvironment and the host's response. In both cases, this raises the question of the effects of microsporidian infection at the host and host-cell levels.

We aimed to address the question of human host intracellular response to microsporidian infection through a transcriptomic kinetic study of human foreskin fibroblasts (HFF) infected with A.algerae, a human infecting microsporidia with an exceptionally wide host range. We focused solely on host response studying both coding and small non-coding miRNA expression.

Our study revealed a generalized down-regulation of cell miRNAs throughout infection with up to 547 different miRNAs downregulated at some timepoints and also transcriptomic dysregulations that could facilitate parasite development with immune and lipid metabolism genes modulation. We also hypothesize possible small nucleic acid expropriation explaining the miRNA downregulation.

This work contributes to a better understanding of the dialogue that can occur between an intracellular parasite and its host at the cellular level, and can guide future studies on microsporidian infection biology to unravel the mode of action of these minimalist parasites at the tissue or host levels.We have also generated a kinetic and comprehensive transcriptomic data set of an infectious process that can help support comparative studies in the broader field of parasitology. Lastly, these results may warrant ***for caution regarding microsporidian exposure and persistent infections.***

## 1 Introduction

Some prokaryotic and eukaryotic parasites have evolved into an obligate intracellular lifestyle using diverse mechanisms to invade and survive in host cells with varying impact on their host's health. The effects range from strong pathogenic marks to milder alterations of host and host cell functions [1]. Microsporidia are a group of obligate intracellular parasites related to fungi and possess some of the smallest known eukaryotic genomes [2,3]. They represent interesting models to explore host-pathogen interactions at the cellular level and can help in giving novel insights both on host cell responses and on intracellular pathogen biology. Indeed, microsporidia can thrive in various environments and can be found infecting virtually all animal species. They are found in food and natural water supplies as spores and infect their hosts through faecal-oral transmission or via contact with a mucosa or an open wound [[4], [5], [6]] [[4], [5], [6]] [[4], [5], [6]]. Microsporidia are responsible for both opportunistic and non-opportunistic infections in their various hosts. Once in contact with cells or tissues, spores can extrude an invasion apparatus called the polar tube towards neighboring cells. The infectious content of the spore, *i.e.* sporoplasm, will transit through the extruded polar tube until reaching a host cell's cytoplasm. The sporoplasm then quickly enters a proliferative phase, the merogony, followed by a differentiation phase, the sporogony, which will lead to the formation of new spores inside the host cell. Spores will finally be released either through lysis of the cell or active secretion (exocytosis) depending on the microsporidian specie [7,8].

In humans, microsporidian infections predominantly occur in the digestive tract. Although infections are mainly asymptomatic, they can be much more critical in immunocompromised patients and induce enteropathies, myositis, pneumonitis, prostatitis, urethritis, or even disseminated sepsis [9].

Both symptomatic and asymptomatic microsporidian infections may persist in individuals for several months or even years in some cases [10]. Those persistent infections are a topic for concern given the opportunistic nature of microsporidia and how widespread they are in the environment [6,10,11]. To add to this concern, stressors associated with modern life like pollution, pesticides and biological agents have shown immunomodulatory capabilities, thus making opportunistic infections and co-infections a crucial theme in modern research [[12], [13], [14]]. A recent publication has also identified latent microsporidian infection as a risk factor in colon cancer patients [15]. However, apart from such recent reports the potential health concerns a persistent microsporidian infection may present has not been explored much.

Humans are found infected by six main genera of microsporidia namely *Enterocytozoon*, *Encephalitozoon*, *Anncaliia*, *Vittaforma*, *Trachipleistophora*, and *Pleistophora* [9]. Among them, the species *Anncaliia algerae* is an oddity as it can infect both humans and the phylogenetically distant anopheles insect species. *In vitro*, *A. algerae* has also shown great ability in infecting and proliferating in many different cell types derived from mammalians, fish and insects [9,16].

Transcriptomic approaches have been used in the context of various host-pathogen interaction studies and have helped gain a better overview of the processes at play during this dialogue. Through the identification of modulated genes and their associated functions and pathways, researchers have gained insight on the host-pathogen interactions of viruses [17,18], intracellular parasites like *Toxoplasma gondii* [19,20], *Plasmodium falciparum* [21] and more, as well as the cellular response of their hosts [19,22].

Through transcriptomics it is also possible to study the roles of microRNAs in host-pathogen interactions. MicroRNAs (miRNAs) are a class of small non coding RNAs (ncRNAs), typically 21–25 nucleotides long, involved in translational repression, degradation, and overall regulation of messenger RNAs (mRNAs) [23]. They are involved in numerous physiological and pathological processes, and their role in host-pathogen interactions is being increasingly studied and has been demonstrated in several infection models such as *Trypanosoma* infection, *Leishmania* infection, *Toxoplasma* infection and counting [24].

Despite this, only few transcriptomic studies on microsporidian species have researched host response at the cellular level integrating both small RNA and mRNA data for a more complete overview of the transcriptomic response of the host cells. There is also a lack of kinetic data following the whole infectious cycle.

We therefore studied host responses to *A. algerae* during its whole parasitic cycle in the *in vitro* infection model of human foreskin fibroblast (HFF) cells. We integrated both mRNA and miRNA transcriptomic data, focusing on host gene expression, aiming to decipher the dialogue that takes place between human cells and the pathogen.

This effort yielded evidence of host cell transcriptomic dysregulation following *A. algerae* infection and highlighted an important and seemingly aspecific down-regulation of host miRNAs from 12h to 72h post-infection.

## 2 Results/discussion

### 2.1 The human cell transcriptomic profile induced by *Anncaliia algerae* infection is characterized by a general miRNA down-regulation

#### 2.1.1 Infected cell's transcriptomic profile is strongly marked by miRNA down-regulation

**Fig. 1:**
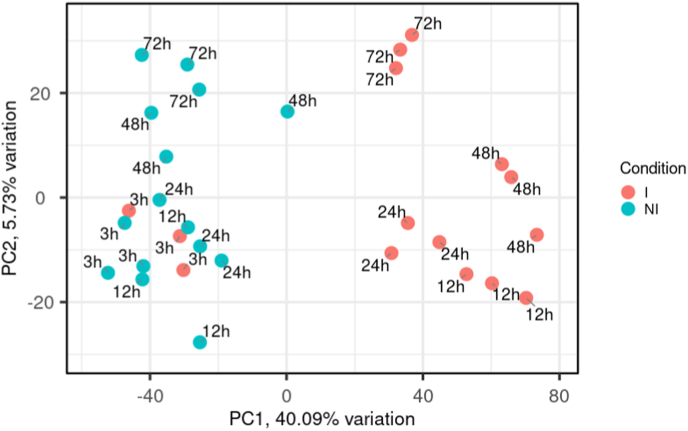

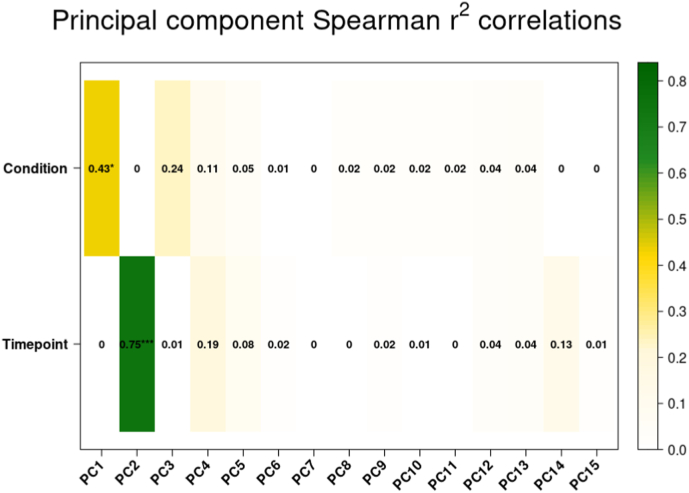
Principal component analysis (PCA) of infected (I) and non-infected (NI) samples. **A.** PCA plot of each sample along principal components 1 and 2 (PC1, PC2). Blue dots represent non infected samples (NI); Red dots represent *A.algerae* infected ones (I). For each dot the corresponding time-point is indicated. The first principal component (PC1) and the second one (PC2) explain 40.09% and 5.73% of the overall variance respectively. **B.** Plot of Spearman r^2^ correlation between each of the first 15 principal components and the infectious status (I or NI) or time (time-point in the infection kinetic). Stars represent significance after correlation test with p-value ≤ 0.001 (*) and p-value ≤ 0.00001 (***). Results show that PC1 correlates with infection condition (R^2^ 0.43*) while PC2 correlates with sampling time (R^2^ 0.75***) **C.** Histogram showing the proportion of miRNA and protein-coding genes that contributed more to PC1 than to any other principal component.

To study the global impact of *A. algerae* infection on the human cell transcriptome, a Principal Component Analysis (PCA) was conducted on the transcript count data of both mRNAs and miRNAs for each sample (Fig. 1 A). Samples corresponded to 3h, 12h, 24h, 48h and 72h post-infection (pi) timepoints. This spanned over a whole life cycle of *A.algerae*. An unambiguous separation between infected (I) and non-infected (NI) conditions following PC1's axis was observed for each time-point except for the 3h pi samples which clustered together on the NI samples' side. This result suggests distinct changes in host transcriptomic profile between 3h and 12h post *A.algerae* infection, and PC1 contributing genes seem to be the main drivers.In contrast, PC2 seems to separate samples by time-point, and no difference was observed between I and NI samples along this axis suggesting that this distribution is due to host cell life cycle progression independently of the infection stage.

We further analyzed the association between the PCs and the experimental variables, *i.e.* pi time and infection status, through a Spearman R^2^ correlation test (Fig. 1 B). This test confirmed correlation between infection status and PC1 (R^2^ 0.43 p-value ≤ 0.01) and revealed strong correlation between PC2 and time-point (R^2^ 0.75 p-value ≤ 0.0001), all in agreement with our observations. PC1 and infection status correlation value is likely diluted by the 3h infected samples as they clustered with the non-infected samples.

To identify the genes specifically driving the differences in transcriptomic profiles between I and NI samples, analysis of the PC1 loadings was conducted. All genes with a higher contribution to PC1 than to any other principal component were then selected. Interestingly, an overwhelmingly high proportion of miRNA genes with 257 against only 1 protein-coding gene (the POLA1 gene, DNA polymerase Alpha 1 catalytic subunit) was observed (**S1 File**). This analysis reveals that the down-regulation of a large number of miRNAs and the upregulation of POLA1 seem to be strong features driving the transcriptomic profile of host cells throughout *A.algerae* infection.

#### 2.1.2 miRNA down-regulation seems to be generalized and independent of pi time-point while protein-coding gene dysregulation seems more time-point specific

**Fig. 2:**
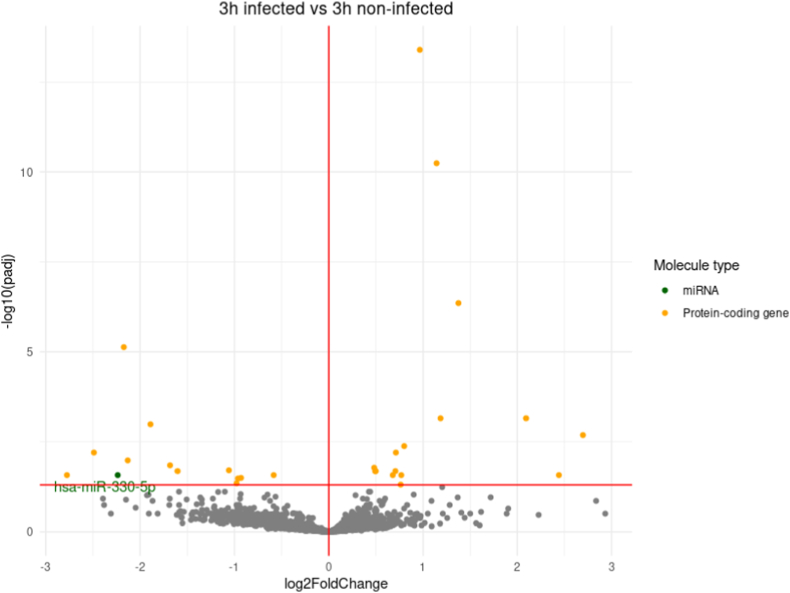

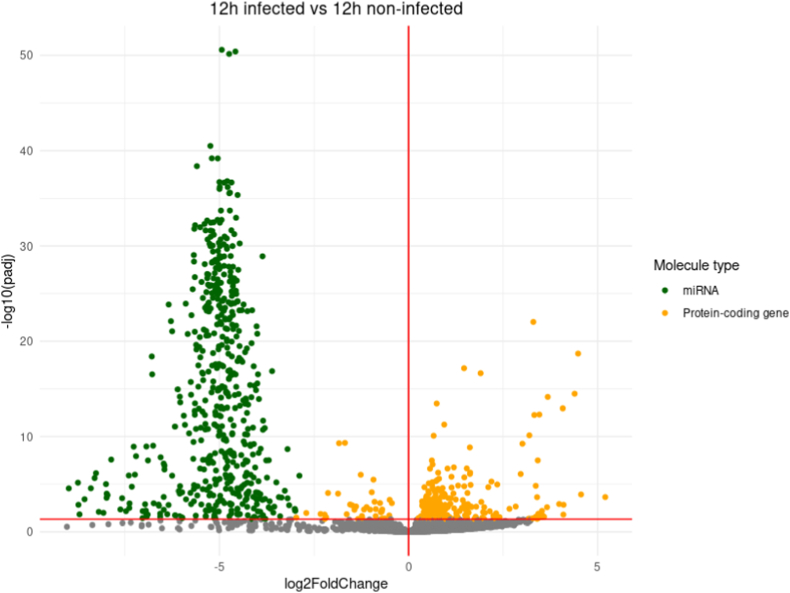

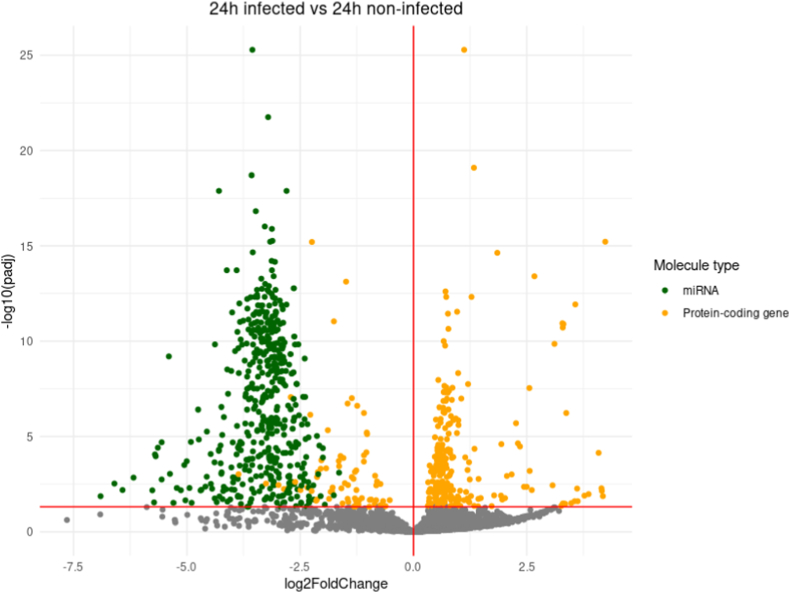

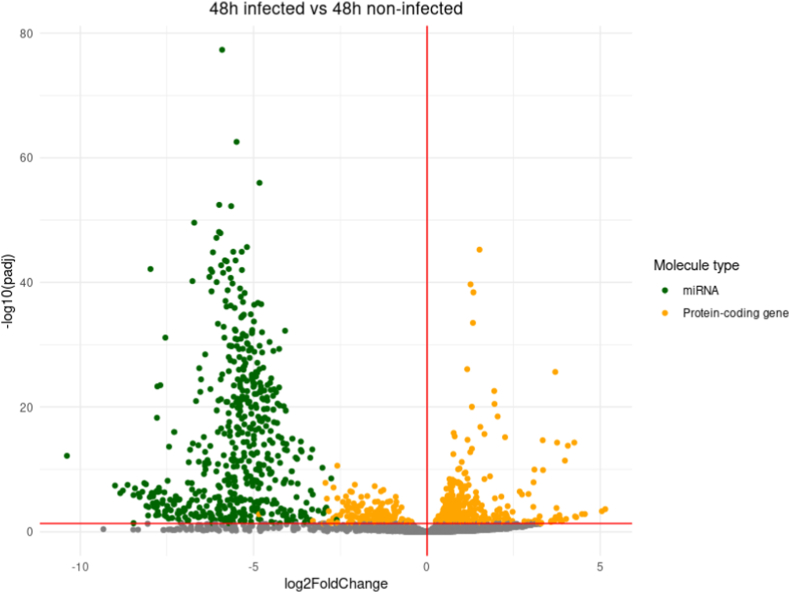

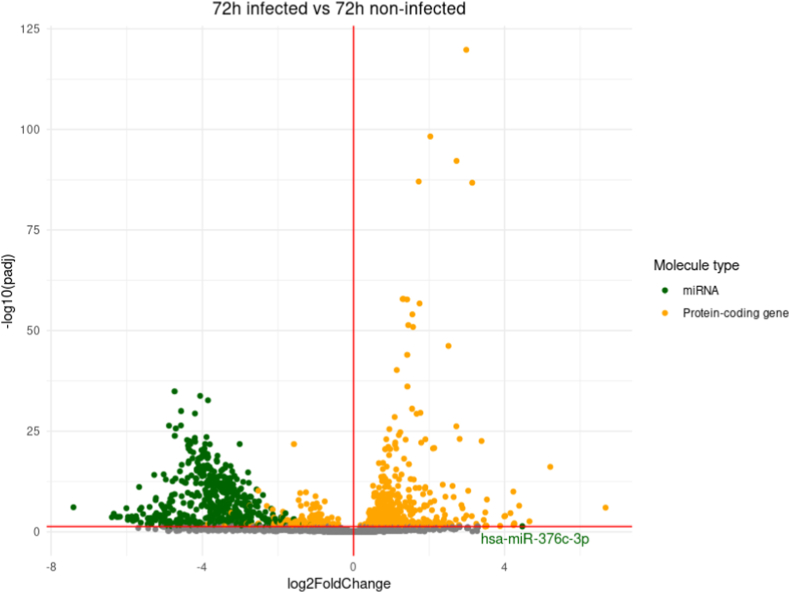

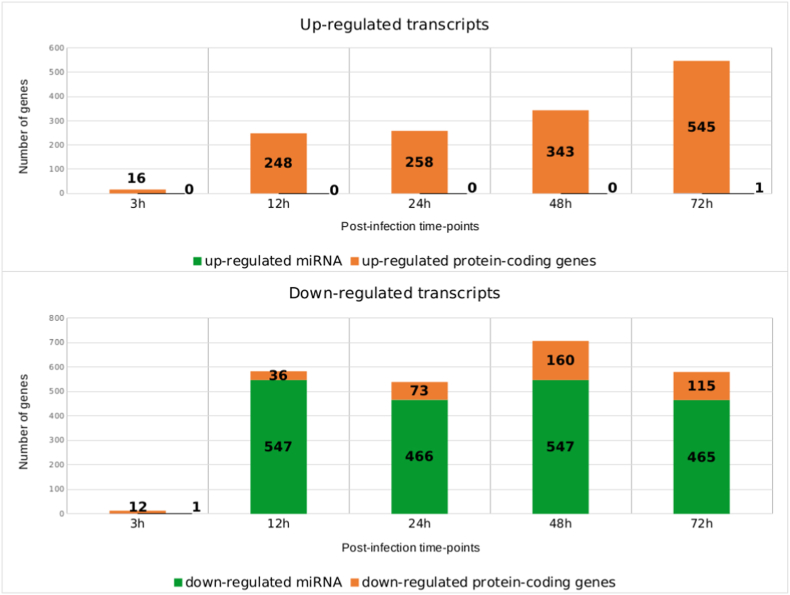

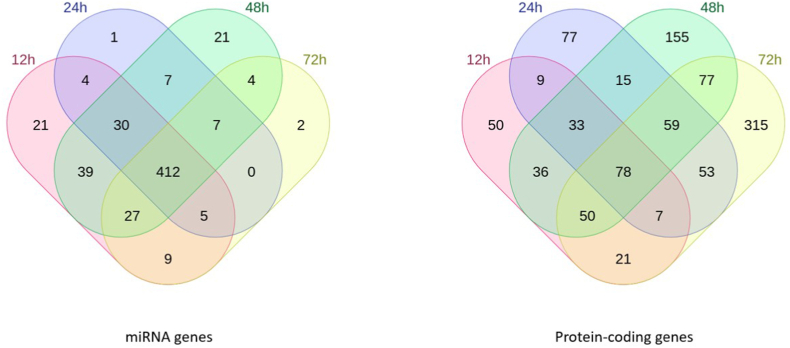
Differential expression analysis (DE) between infected (I) and non-infected (NI) conditions. Volcano plot of differentially expressed genes between I and NI(control) samples for each time-point, respectively 3h (A), 12h (B), 24h (C), 48h (D) and 72h (E) pi. Log2fold change is plotted in x-axis and adjusted p-value in ordinate. Each dot corresponds to one transcript in our data. miRNA genes with adjusted p-values under 0.05 are highlighted in blue. **F.** Histograms of the number of up and down-regulated miRNA and protein-coding genes. **G.** Venn diagram of the dysregulated miRNA genes and protein-coding genes at different timepoints. DE analysis of the 3h pi samples was discarded for this analysis.

A differential expression analysis comparing transcript expression between infected and non-infected conditions for each time-point was conducted (Fig. 2). The overall trend is to a higher number of down-regulated genes than up-regulated ones with for example 583 down-regulated genes at 12h pi and only 248 up-regulated ones at the same time-point. The vast majority of down-regulated genes correspond to miRNAs, and only one miRNA gene, hsa-miR-376c-3p involved in G1-cell cycle arrest [25,26], is found up-regulated in the whole kinetic (Fig. 2 A-F).

Up to 547 miRNA genes were found down-regulated at once (12h and 48h pi time-points), representing about 20.6% of the 2653 miRNAs present in our count matrices (**S2 File**). This percentage would likely be even higher if we only considered the 2300 true human miRNAs as estimated by J.Alles *et.al.* [27].The average log2 fold change of the down-regulated miRNA genes is about −4 over the whole kinetic. Interestingly, these log2 fold changes also seem to draw a gaussian-like shape for most time-points which could suggest an aspecific or generalized process of miRNA down-regulation. Looking at Venn diagrams of the dysregulated genes between 12h, 24h, 48h and 72h pi time-points (Fig. 2 G), we can see that most dysregulated miRNA genes are common throughout the infectious process with 412 common in the total 589 dysregulated miRNA genes (about 70%). Meanwhile, most of the protein-coding gene dysregulation is time-point specific with only 78 out of the 1035 being common (about 7.5%).

All this together suggests that the observed miRNA dysregulation, unlike the protein-coding gene dysregulation, could be a generalized process, not specific to a uniquely defined subset of miRNAs or to a certain time-point.

Such generalized miRNA down-regulation has not been described in other parasites or microsporidia to our knowledge. For microsporidia this could be attributed to the fact that within the few studies that accounted for small non coding RNA, most were carried out at the whole organism or tissue levels rather than the cellular level [28,29]. In those cases, the response observed stemmed from multiple tissues and cell types and could render the miRNA down-regulation undetectable. There was one study however which was carried out on *Bombyx mori* embryonic cell line describing their response to the microsporidian *Nosema bombycis* [30]. Nonetheless global miRNA expression was not the main scope of the study and miRNA counts were normalized using Transcripts Per Kilobase Million (TPM) before differential expression analysis thus giving miRNA abundances relative to one another. This would make a generalized down-regulation of miRNA undetectable when comparing samples [31]. Only 1 sample had been published per condition in the study and so we could not re-analyze the data using our methodology. Regardless, it is also possible that the massive miRNA down-regulation we observed here is specific to *A.algerae* infection, or to a defined subset of microsporidian species.

Search for a common transcription factor network governing all down-regulated miRNAs using Transcription factor enrichment analysis using Transmirv2.0 [32] did not reveal any candidates (S1 Table). This led us to hypothesize that the miRNA down-regulation may occur through parasite action on already transcribed miRNAs.

#### 2.1.3 Parasite activity could explain host miRNA down-regulation throughout infection

To explore possible causes for the miRNA downregulation, a correlation analysis of gene expression throughout the infection cycle was executed between the downregulated miRNA genes and both host and parasite protein-coding genes. Focus was put on protein-coding genes whose expression was negatively correlated with the down-regulated miRNAs as they may be involved in the process.

**Fig. 3:**
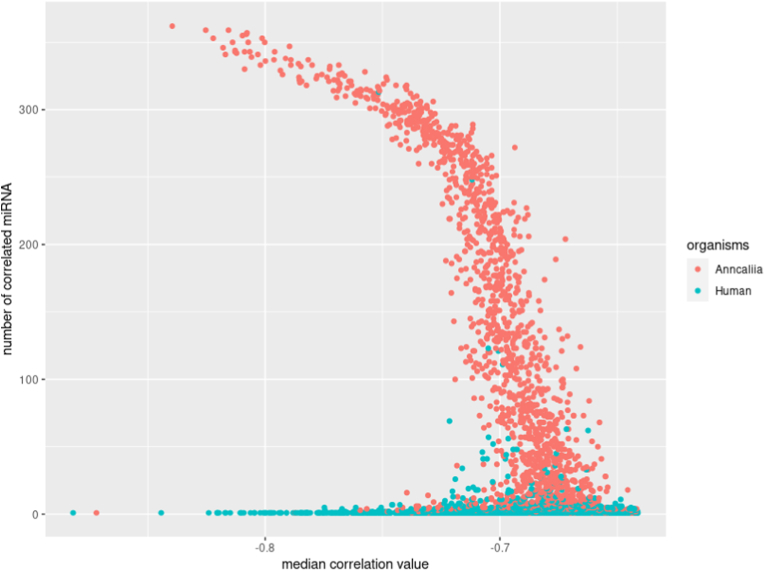
Small RNA size distributions between infected and non-infected samples and correlation analysis of coding gene expression and host down-regulated miRNA. Plot of the spearman correlations between the expression level of *A.algerae* genes and the number of down-regulated host miRNAs. A p-value cut-off was set at 0.01 and only negative correlations were considered.

Interestingly, we found that the genes that correlated the most and with the most miRNAs were all *A.algerae* coding genes (Fig. 3).

We further looked at the top protein-coding genes and identified their function when possible through NCBI conserved domain search and BLASTP analysis (S2 Table). Of note in this list is the presence of 4 transport associated genes, 7 cell growth proliferation and development associated genes as well as a 6-phosphogluconate deshydrogenase involved in the pentose phosphate pathway leading to ribonucleotide synthesis [33] and also a trehalose phosphate synthase, involved in the production of threalose-6-phosphate and UDP from UDP-glucose and glucose-6-phosphate [34].

Given the most correlated genes pertain to pentose phosphate pathway, transport and proliferation associated genes, we hypothesize a possible import of host small nucleic acids into the parasite followed by degradation and use as substrate for its own proliferation and nucleic acid synthesis. These results call for dedicated exploration.

#### 2.1.4 A higher-than-average portion of the up-regulated protein-coding genes are predicted targets of the down-regulated miRNAs

**Fig. 4:**
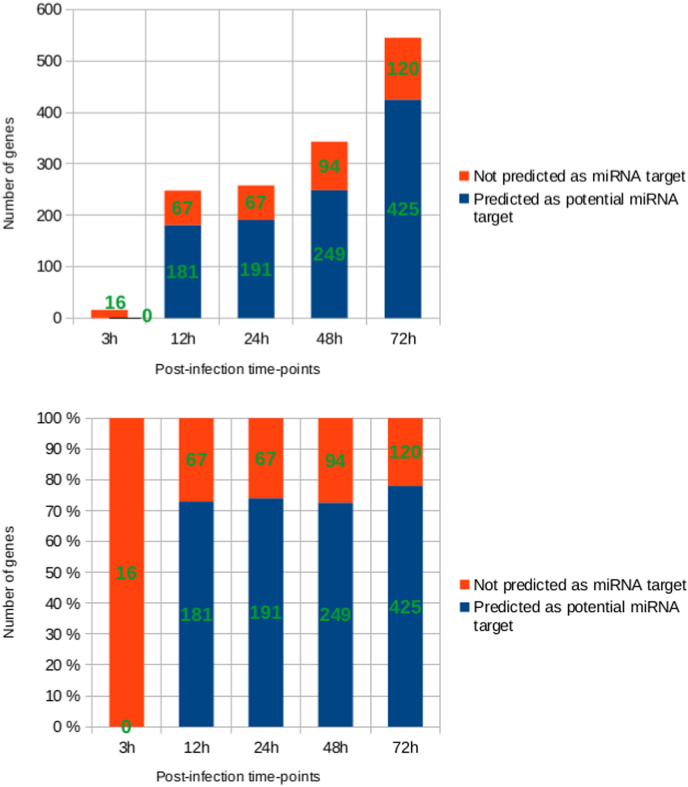

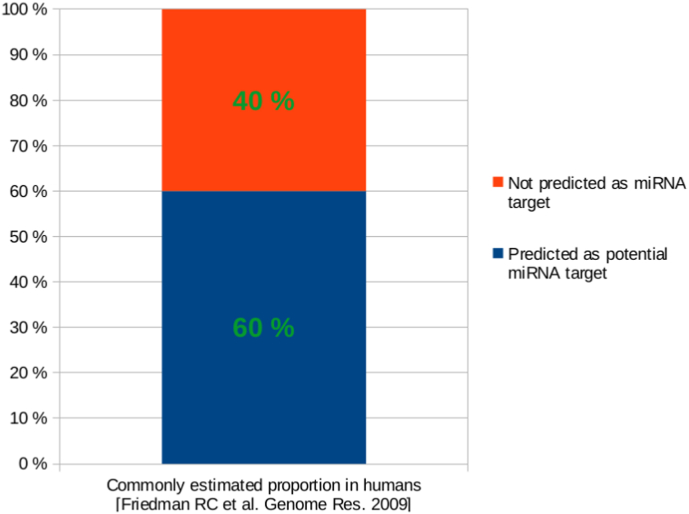
Proportion of up-regulated genes predicted as targets of the down-regulated miRNA. **A.** Histograms of the number and percentages of up-regulated protein-coding genes predicted as targets of down-regulated miRNA, for each post-infection time point. **B.** Percentage of human protein-coding genes under miRNA control based on the study by Friedman RC et al. [35]. This number was estimated using phylogenetic conservation of miRNA target sites as one of the criteria for validation. For the sake of comparability, our predictions were also done using databases which implement this aspect (i.e. miRanda, pita and targetscan).

Regardless of the cause behind their down-regulation, miRNAs remain key regulators of gene expression. We wondered whether the downregulation of miRNAs in our experiment could directly contribute to the observed up-regulation of some protein-coding genes. Indeed, miRNAs are mainly described as mRNA repressors and so their down-regulation could lead to the up-regulation of their direct target transcripts. Hence, the percentage of up-regulated protein-coding genes predicted as targets of the down-regulated miRNAs was estimated at each post-infection time-point (Fig. 4 A). A comparison of these values with the average number of protein-coding genes under miRNA regulation in humans as estimated by Friedman RC *et al* [35] (Fig. 4 B), showed a higher than average proportion of protein-coding genes under down-regulated miRNA control throughout infection. Percentages were above 70% even reaching 78% at 72h pi which is higher than the average of about 60%.This would suggest that the miRNA down-regulation could be reinforcing the protein-coding gene dysregulation inside host cells throughout infection by *A.algerae*.

**Fig. 5:**
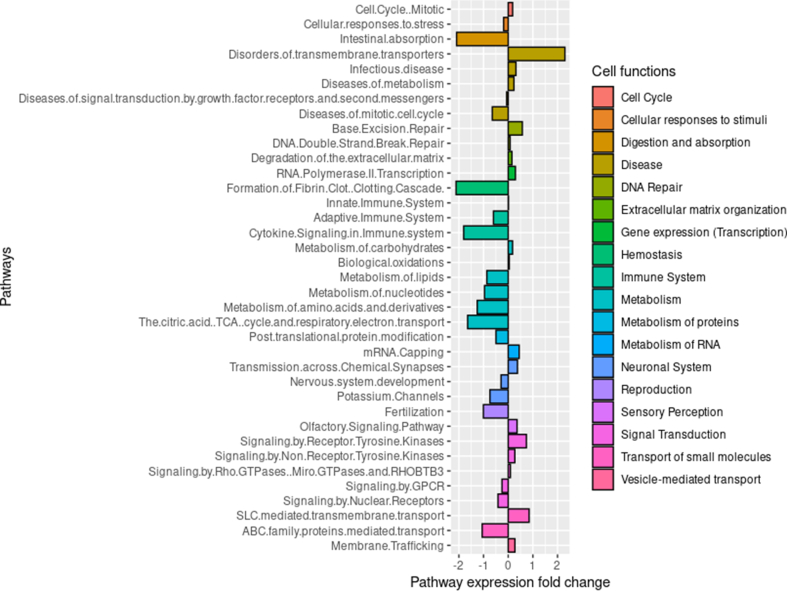
Gene set enrichment analysis (GSEA) of protein-coding genes and miRNAs. Average fold change of pathways and processes between infected (I) and non-infected (NI) conditions as calculated by Reactome CAMERA GSEA. Multiple cellular pathways are found either up or downregulated.

Next, to investigate the functional relevancy of the protein-coding gene dysregulation in human cells and to estimate the contribution of the down-regulated miRNA targets in the overall effect, a gene set enrichment analysis (GSEA) of the whole transcriptomic expression data was done. Then, contribution of miRNA targeted genes in the enriched pathways was estimated. GSEA was done comparing infected and non-infected conditions, using 12h, 24h, 48h and 72h data points combined, to focus on dysregulation trends that persisted throughout infection by *A.algerae* (Fig. 5).

Results showed enrichment of genes related to immune system, hemostasis, extracellular matrix organization, vesicle-mediated transport, transport of small molecules, digestion and absorption, general metabolism, protein metabolism, RNA metabolism, cell cycle, DNA Repair, gene transcription, neuronal system, reproduction, sensory receptors, and signal transduction. Some pathways that were identified were not relevant to the cell type in our experiment as they pertained to other specialized cell types and were thus later discarded (olfactory pathways, neuronal pathways, and reproduction). The main down-regulated pathways were associated with adaptive immunity, cytokine signaling, formation of fibrin clot, ABC family mediated transport, intestinal absorption, lipid metabolism, nucleotide metabolism, amino acids and derivatives metabolism, citric acid TCA cycle and respiratory electron transport, as well as G protein-coupled receptor (GCPR) signaling and signaling by nuclear receptors. The main up-regulated ones were Solute carrier family proteins (SLC) mediated *trans*-membrane transport, membrane trafficking, degradation of the extracellular matrix, metabolism of carbohydrates, mRNA capping, base excision repair, cell cycle mitotic, RNA polymerase II transcription, signaling by receptor tyrosine kinase and non-receptor tyrosine kinases.

The proportion of down-regulated miRNA targets in the up-regulated pathways was then calculated. Results show weak and uneven presence of the miRNA targets in the different dysregulated pathways (S3 Table). This suggests that the miRNA down-regulation is likely not the main driver behind the dysregulation of host pathways identified by our GSEA. On the other hand, the miRNA down-regulation in itself may have other implications for human cell physiology as some of the miRNAs found down-regulated are known to be associated with key functions like immune responses (e.g. hsa-miR-21, hsa-miR-155), cytokine-mediated apoptosis (e.g. hsa-miR-30b), or senescence processes (e.g. hsa-miR-24) [24]. Their down-regulation could participate in allowing *A.algerae* proliferation, development and overall survival (**S3 File**).

### 2.2 *Anncaliia algerae* induces transcriptomic dysregulation that could be beneficial to parasite development through impact on various cellular pathways

#### 2.2.1 Dysregulation of various immunity, lipid metabolism and DNA repair pathway-related genes throughout *A.algerae* infection

We sought to better understand the host pathway dysregulation during *A.algerae* infection through a more in depth analysis of our GSEA results. This analysis revealed relatively weak signs of host cell defense system activation and an overall trend of parasite benefiting changes.

First, some immunity-related pathway genes were observed as perturbed (**S4 File**). Our data revealed up-regulation of host STING, and STAT6 chemokine induction pathways. The STING pathway is involved in cytosolic pathogen DNA detection, and chemokines play an important role in inflammatory responses [1], this could translate activation of some host cell defense mechanisms. Nevertheless, these seem to be the only enriched immunity related pathways upregulated in our experiment. Some studies have reported strong but diverse transcriptional responses to microsporidan infections between nematodes, insects, and vertebrates. These studies reported anti-microbial peptide (AMP) upregulation in silkworms infected by *Nosema bombycis* and cytokines IL2-STAT5, IL6–JAK–STAT3 and ROS upregulation in *Encephalitozoon intestinalis*-infected human enterocyte cultures [36,37]. Interestingly, in our data the only other antimicrobial and/or cytokine pathways related genes found upregulated were ATP7A (MNK) and SLC11A1 (Nramp1).

Both ATP7A and SLC11A1 code for metal ion transporters.ATP7A transports Cu(I) and SLC11A1 transports iron and manganese [38]. Outside of their known role in the resistance to some infections through metal ion transport and concentration, these transporters could also be facilitating parasite acquisition of essential metal ions from the host as previously reported with *Neisseria* and *Mycobacterium* host transferrin and lactoferrin hijacking [[39], [40], [41]], or with Nramp1 and *Salmonella*, where host Nramp1 upregulated pathogenicity island-2 virulence genes through iron responsiveness [42,43]. Microsporidia possess iron–sulphur cluster assembly proteins in their mitosomes [44] and host derived metal ions could be important for microsporidian metabolism and/or protein synthesis.

Conversely, several immune-related pathways are found down-regulated.Among them, the IL18 pathway is notable. Our data revealed down-regulation of IL18RAP and ALOX5 genes. ALOX5 codes for a leukotriene synthesizing enzyme involved in inflammation and immune responses [38] and IL18RAP produces an accessory protein required for NF-κB and MAPK8 activation upon IL18 binding. IL18 is involved in Th1 cell and NKT cell activation [45]. Down-regulation of these genes in both immune cell types and non-professional immune cells such as the fibroblasts used in our experiment could lead to impairment of adaptive cell mediated immunity required for intracellular pathogen clearance [1,46].

Two other adaptive immunity related functions found to be down-regulated are linked to lymphoid and non-lymphoid cell interaction, and to antigen cross presentation.Antigen cross presentation is a mechanism by which some antigen presenting cells capture and present extracellular antigens on their MHC I receptors to CD8^+^ T cells [47]. The downregulation of these genes could have repercussions on the fibroblast's ability to present antigens on its MHC I and thus impair CD8^+^ T cell detection and clearance of infected cells. This could promote persistence of infection as adaptive immunity has been shown to be central in anti-microsporidia immunity, parasite control, and clearance [46].

Other classes of host function found dysregulated in our *A.algerae* infection experiment are small molecule transport and vesicle mediated transport (**S4 File**).GSEA revealed a down-regulation of multiple ABC family transporters involved in lipid homeostasis (namely ABCG, ABCA, ABCD), and the PEX19 protein. ABCG gene products are known to participate in the export of cholesterol out of enterocytes [38] and are also involved in biliary export of sterols. ABCA family members participate in the transport of various molecules across organelle membrane and cell plasma membrane [38]. Additionally, they may also have roles in macrophage lipid metabolism. ABCD are involved in peroxisomal import of both fatty acids and acylCoAs, whereas PEX19 is a chaperon protein involved in peroxisomal function [38]. The down-regulation of all these genes may contribute to higher cytoplasmic availability of cholesterol and fatty acids, making them available for use by the parasite for its own energy metabolism and membrane development [[48], [49], [50]]. In addition, ABC transporters are ATP-dependent and it should be noted that their down-regulation can also reduce ATP expenditure by the cell making more of it available for use by the parasites. It has been shown that microsporidia can import host ATP to ensure their intracellular development cycle through specific parasite coded ATP transporters [51]. Since microsporidia only possess a relic version of mitochondria (*i.e.* the mitosome, incapable of oxidative phosphorylation) this strategy is thought to be crucial for these parasites to gather the necessary energy for proliferation and development. The down-regulation of host ABC transporters would seem to present a double advantage for the parasite which can thus benefit from more abundant lipid and energy resources to ensure its life cycle. Several metabolic pathway related host genes were also dysregulated as we can expect following an intracellular parasite infection (**S4 File**).

Concerning mitochondrial metabolism, numerous genes associated with mitochondrial uncoupling processes, which produce heat by shunting the H+ gradient required for ATP production [52], were found to be down-regulated. This down-regulation could lead to higher ATP production and availability in the host cell hence benefiting the pathogen as mentioned before. The two uncoupling genes downregulated in our data are SLC25A14 and PM20D1. They respectively code for an uncoupling protein (UCP) and a peptidase involved in the production of N-fatty acyl amino acids from free fatty acids and amino acids [38]. N-fatty acyl amino acids themselves function as mitochondrial uncoupling molecules with various impact on cell physiology [53]. Lower PM20D1 activity could also result in higher pools of free fatty acids and amino acids in the cytoplasm of host cells, leaving them available for parasite import and use.

Pyrimidine catabolism, phosphatidyl ethanolamine (PE) and phosphatidyl serine (PS) acyl chain remodeling related genes were also down-regulated, along with genes related to intestinal lipid absorption and digestion. Regarding PE and PS acyl chain remodeling, work from El Alaoui *et. al.* [54] showed the existence of a bacteria- and fungi-typical pathway for phospholipid synthesis in microsporidia. They have also highlighted higher activity of phosphatidylserine decarboxylase and phosphatidylethanolamine N-methyltransferases in cells infected by the microsporidian *E. cuniculi*. Combined activity of these enzymes can lead to the conversion of phosphatidylethanolamine and phosphatidylserine into phosphatidylcholine. Another more recent study in drosophila has revealed phosphatidic acid as a limiting host metabolite for the proliferation of the microsporidian *Tubulinosema ratisbonensis* [55]. Based on these observations, it can be hypothesized that the down-regulation of PE and PS acyl chain remodeling associated genes, could translate a reduction of cellular PE and PS through their transformation by phosphatidylserine decarboxylase and phosphatidylethanolamine N-methyltransferases into phosphatidylcholine, which could play an important role in *A.algerae* development and proliferation. Our GSEA also showed choline catabolism pathway down-regulation which further supports this hypothesis.

Interestingly, phosphatidylcholine has been described as essential for both *Plasmodium berghei* and *P. falciparum* development and survival in hepatocytes [56], and several reports indicated that microsporidian infections in anopheles can impair *Plasmodium* transmission suggesting of an antagonism between microsporidian and *P.falciparum* [[57], [58], [59], [60], [61]].

Concerning the down-regulated genes linked to intestinal lipid absorption and digestion, The NPCL1L1 gene is of special interest. It codes for a transporter allowing enterocyte uptake of free cholesterol and phospholipids for subsequent transport towards the general circulation in humans [62]. Thus, such a down-regulation could lead to a digestive micro-environment with higher amounts of lipids but could also mean enterocyte cells with lower amounts of them. Whether this alteration benefits the parasite remains unclear.It is also conceivable that this down-regulation is a consequence of high concentrations of intracellular lipids in the cytoplasm, namely polyunsaturated fatty acids. This kind of down-regulation has been previously reported *in vitro* as well as in the case of high cholesterol diets in animals [62,63].

The intracellular development of *A.algerae* also has an impact on genes involved in DNA repair pathways (**S4 File**). More specifically double stranded break repair genes, including homologous recombination (HDR) and base excision repair (BER) genes, were found to be up-regulated. GSEA also shows slight up-regulation of numerous genes involved in Tumor suppressor protein p53 (TP53) protein activity (**S4 File**). A study on *E.intestinalis* reported a higher mutation rate of microsporidia infected cells with up to 2.5 times that of non-infected cells [64]. Authors of the paper hypothesized oxydative stress caused by infection to be behind this phenomenon as free radicals and reactive oxygen species (ROS) may induce nicks and damages in DNA. Our data further reinforces this hypothesis as BER is described as the main repair mechanism to free radicals-induced DNA damage [65] and there have also been reports of a type of HDR being activated following DNA nicks [66]. TP53 related gene activation may also due to these potential DNA nicks as TP53 plays a central role in orchestrating DNA repair [67].

#### 2.2.2 Dysregulated disease-associated genes suggest the presence of exogenous RNA in host cell and/or transposable element mobilization

Throughout *A.algerae* infection the human cell transcriptome also showed dysregulation of some genes with documented disease associations. GSEA revealed metabolic, signal transduction and infectious disease related genes (Fig. 5). Interestingly, all infectious disease related genes were upregulated and were related to those involved in infection processes by the Human Immuno-deficiency Virus (HIV): Transcription of the HIV genome and RNA polymerase II subversion by the HIV were the two enriched functions (**S4 File**). GSEA also revealed up-regulation of RNA polymerase II transcription genes which may be linked to the same response (**S4 File**).The infectious disease databases tend to be diversely annotated in terms of pathologies and pathogens [68], therefore the fact that only HIV-related processes were found enriched in the infectious disease related pathways is most likely not an artifact and would suggest some similarities in host response to both infections.

An explanation for this could be the presence of *A. algerae* retrotransposons in the host nucleus. Indeed, genomic studies have shown that *A.algerae* presents a very high diversity of transposable elements (TE) in its genome with over 240 TE families [69]. Phylogenetic analysis of these TEs has revealed that some of them most likely came from recent horizontal and bidirectional transfers with metazoans [69]. Such transfers may also induce DNA repair mechanisms like HDR which would be coherent with our results.

Finally, there have also been reports of parasites acting as vectors of viruses [70]. Given the large diversity of TEs in *A.algerae* and it's wide host range, it is possible that *A.algerae* could also act as a vector for degenerated pieces of lysogenic viruses.

A study in *Caenorhabditis* nematodes infected by *Nosema parisii* and *N.ausubeli*, two other microsporidian species harboring TE in their genomes [71], also reported host responses with similarities to *C. elegans* infection by two RNA viruses [72].

## 3 Conclusion

To conclude, we highlighted a previously unreported massive down-regulation of host miRNAs throughout *A.algerae* infection. These results call for further study of the effects of microsporidian infections, especially in the context of co-exposition with other stressors given the major role miRNAs have in physiological and pathological processes. This work may already help in explaining the existing reports of adverse effects of microsporidian co-exposure in diseases [15,73]. Whether this down-regulation occurs in other microsporidian infections remains to be explored and demonstrated. Interestingly, work on honeybees infected by the microsporidia *Nosema ceranae* revealed that in the presence of siRNA mediated knockdown of parasite dicer protein, host miRNA dysregulation was abolished. This study was done on bee mid-guts [74]. Although organ and cell level responses may vary, their results could suggest that all dicer expressing microsporidia may have an impact on host miRNA expression.

In our study, the observed transcriptional dysregulations also corroborates previous studies on microsporidia infection biology and impact on organisms [9,37,48,49,[75], [76], [77]]. Namely, our data further suggests a role of phosphatidylcholine in microsporidian biology, the downregulation of some immune pathways, and the induction of DNA damage and higher mutation rates in infected cells.

The general cell pathways dysregulated did not strongly vary throughout the infectious cycle (from 12h to 72h post-infection).

Finally, our results also revealed a host response that could hint at TE activity and/or exogenous RNA presence throughout infection. Whether other human infecting microsporidia induce similar responses remains to be explored.

Comparison *A.algerae and* an *Encephalitozoon* infection would be especially interesting as they are from 2 distinct genera and transposable elements are also lacking in *Encephalitozoon* genomes. This could clarify the role of parasite derived TEs in host responses.

## 4 Materials and methods

### 4.1 *Anncaliia algerae* spore production, infection kinetics in human foreskin fibroblasts, and RNA extraction

*Anncaliia algerae* Undeen strain (reference ATCC PRA-339™) was propagated *in vitro* in Human foreskin fibroblasts cells (HFF) (reference ATCC SCRC-1041) for spore production and were cultured in minimum essential medium (MEM) supplemented with 50 μg/ml Gentamicine 1% Amphotericine B, 1% Penicillin/Streptomycin 1% Glutamine and 10% Foetal bovine serum in 25 cm^2^ culture flasks at 30 °C in 5% CO_2_ atmosphere.

For the experiment, initial Non-infected HFF cells were grown in the same conditions as above but kept at 37 °C.

Infection was performed once the HFF cell culture were at about 80% confluence. Half of the 50 culture flasks were each infected with 8.82.10^^6^ spores of *Anncaliia algerae*, which roughly corresponds to an MOI of 4.

Briefly, spores diluted in culture media were added to the cell cultures to a final volume of 5 ml. Flasks were incubated at 30 °C for 1h then rinsed with fresh media. Cultures were kept at 30 °C from that point on until extractions. The 25 remaining flasks were kept as uninfected controls and were also incubated at 30 °C like the others.

Later, 5 uninfected controls and infected cell cultures were lysed with 1 ml Trizol reagent at 3h, 12h, 24h, 48h and 72h post-infection. Total RNA was extracted from each flask following Trizol reagent (Invitrogen) protocol followed by RNeasy column isolation (Qiagen) according to the manufacturer's protocol and using two columns per flask. For each experimental condition the triplicates with the highest RNA concentration were sent for sequencing.

### 4.2 PolyA+ and small RNA sequencing, pre-treatment, treatment, and QC

Total RNA extractions were sequenced and pre-treated by Fasteris, Life Science Genesupport SA (Switzerland). Both PolyA + RNAseq and small RNAseq were performed on each of the samples. Sequencing was done using Illumina NovaSeq 6000 with NovaSeq Control Software 1.7.0, RTA v3.4.4 and bcl2fastq2.20 v2.20.0.422 as the basecalling pipeline.

For the PolyA + RNAseq, stranded mRNA libraries of about 100bp per molecule were prepared and paired-end sequencing was done.

For the small RNAseq, small-RNA libraries were prepared after size filtering (sequences <50 nt were kept) then single-end sequencing was performed.

For the PolyA + RNAseq the runs yielded from around 1000 to 29,000 Mb with over 6000 Mb on average per sample, and for the small-RNAseq from 2000 to 8000 Mb with over 2700 Mb on average per sample (**S5 File**).

Adapter trimming and inserts retrieval of small RNA reads was done by Fasteris using Trimmomatic version 0.32. Raw data is available as NCBI Bioproject Accession number PRJNA927239.

### 4.3 Quality control and data treatment

Read QC was checked using the FastQC tool [78]. All samples displayed sufficient quality and relevant QC profiles.

For each sample, reads were aligned using bowtie2 [79] to a fused genome file containing both Human GRCh38 (GenBank accession n° GCA_000001405.28) and *Anncaliia algerae Undeen* (GenBank accession n°GCA_000313815.1) reference genomes. Settings used were –sensitive-local.

Average percentages of mapped and unmapped reads were checked and were similar between all samples (**S5 File**).

Count matrices were generated from the bowtie2 bam outputs using Htseq-count from the Htseq framework [80] with the human GRCh38_latest_genomic gff reference annotation file from NCBI. “Transcript” features were counted for the PolyA + RNAseq data, and “miRNA” features for the smallRNAseq data. htseqcount Parameters used for both were “-s yes -m union -r pos”.

Count matrices were normalized using the DEseq2 R package [81] modified using a Variance stabilizing transformations (VSD) prior to QC checks. For QC, Principal component analysis (PCA) and Hierarchical Clustering Analysis (HCA) plots were generated. PCA plot was generated using the PCA plot R package [82].Plots were used to check that triplicates clustered together and that no sequencing, pre-treatment, alignment, or feature counting bias altered the data structure and made unrelated samples cluster together.

Plots showed no apparent bias linked to the pre-treatment and treatment methodologies applied.

### 4.4 Data analysis

#### 4.4.1 Differential gene analysis

For each sample, miRNA and mRNA count matrices were fused. The result was used for differential gene expression analysis (DE) using the DEseq2 R package [80].Independent filtering as well as Cook's cut off were applied when using the DEseq2 “result” command. Volcano plots of the resulting data were generated using the manhattanly R package [83]. Venn diagrams were generated using molbiotools list comparator [84].

#### 4.4.2 Gene expression correlation analysis

Spearman correlations were computed between the down-regulated miRNA, and both host and parasite coding gene expression data using the rcorr function from the Hmisc R package [85]. *A.algerae* expression data was generated similarly to the human expression data using a CDS annotation file from Peyretaillade *et.al.* [86].

Spearman correlations were calculated based on the Deseq2 normalized counts of infected samples. Only negative correlations with a p-value under 0.01 between down-regulated miRNAs and coding genes following Spearman correlation test were kept. Following this, mean and median correlation values, as well as the number of correlating miRNAs were calculated for each of the coding genes.

#### 4.4.3 MiRNA target prediction

MiRNA target prediction was done using the multimir R package [87] searching « predicted » « hsa » targets. Multimir searches through a comprehensive collection of miRNA-target databases namely diana_microt, elmmo, microcosm, miranda, mirdb, pictar, pita, and targetscan. For our analysis only databases with a target conservation score, namely, miranda, pita, and targetscan were used. The top 20% predicted targets were kept.

#### 4.4.4 Gene set enrichment analysis

Gene set enrichment analysis (GSEA) was performed using the Reactome database and tool suite on the raw fused matrices of mRNA and miRNA counts [68]. I and NI conditions were compared, excluding 3h time-points.

GSEA was done using Reactome's « CAMERA » mode [88]. P value cutoff was set at 0.05. Tables were formed in R using data extracted using the ReactomeGSEA R package [89] and the rbioapi R package Reactome related functions [90].

## CRediT authorship contribution statement

**Reginald Florian Akossi:** Methodology, Validation, Formal analysis, Investigation, Data curation, Writing – original draft, Writing – review & editing. **Fréderic Delbac:** Writing – review & editing. **Hicham El Alaoui:** Writing – review & editing. **Ivan Wawrzyniak:** Conceptualization, Methodology, Validation, Investigation, Resources, Data curation, Writing – review & editing, Supervision. **Eric Peyretaillade:** Conceptualization, Validation, Resources, Writing – review & editing, Supervision, Project administration, Funding acquisition.

## Declaration of competing interest

The authors whose names are listed immediately below certify that they have NO affiliations with or involvement in any organization or entity with any financial interest (such as honoraria; educational grants; participation in speakers’ bureaus; membership, employment, consultancies, stock ownership, or other equity interest; and expert testimony or patent-licensing arrangements), or non-financial interest (such as personal or professional relationships, affiliations, knowledge or beliefs) in the subject matter or materials discussed in this manuscript.
