# Supplementary material for: The intracellular parasite Anncaliia algerae induces a massive miRNA down-regulation in human cells
Source: Noncoding RNA Res. 2023 May 18;8(3):363–75. doi: 10.1016/j.ncrna.2023.05.003 (PMC10238475; doi:10.1016/j.ncrna.2023.05.003)
Supplement: Multimedia component 5 [file mmc5.zip › S5 File/SmallRNAseq/DR_2021-05-18_ANOE-1-12.pdf]

Illumina Services
**Data Report**
ANOE-20210210a

Customer		Fasteris	
Account	Eric Peyretaillade	Project	ANOE-20210210a
		Date / ref.	DR_2021-05-18_ANOE-1-12
		Author	Thibault Robin

Summary

Sequenced libraries	**ANOE-1-12**
Number of libraries	**12**
Sample type	**Total-RNA**
Run ID	**210511_A00902_A**
Number of cycle	**2x50+8+8**
Instrument	**NovaSeq**
Yield(GB)	**33.7**
Quality control	**Within specifications**

Author's comments:

No comment

1 Introduction

DNA or RNA samples are processed using illumina technology to prepare libraries, which are then sequenced on Next-Generation DNA sequencing (NGS) instruments.

The present report describes the pipeline used and provides quality and yield information about the data produced.

Upon request, our bioinformatics team is happy to help you to extract the biologically useful information from your data.

NB: Although the illumina NGS instruments deliver high-quality data, you must obtain independent validation of the data.

Illumina PROPEL certified™

2 Library preparation

Sample name	Sample type	Library ID	Library type	Index *
'I 3h 1.1'	Total-RNA	ANOE-1	Small-RNA	ACTGAT
'I 3h 3.1'	Total-RNA	ANOE-2	Small-RNA	ATGAGC
'I 3h 5.1'	Total-RNA	ANOE-3	Small-RNA	ATTCCT
'I 12h 1.1'	Total-RNA	ANOE-4	Small-RNA	CAAAAG
'I 12h 3.1'	Total-RNA	ANOE-5	Small-RNA	CAACTA
'I 12h 5.1'	Total-RNA	ANOE-6	Small-RNA	CACCGG
'I 24h 1.1'	Total-RNA	ANOE-7	Small-RNA	CACGAT
'I 24h 3.1'	Total-RNA	ANOE-8	Small-RNA	CACTCA
'I 24h 4.1'	Total-RNA	ANOE-9	Small-RNA	CAGGCG
'I 48h 2.1'	Total-RNA	ANOE-10	Small-RNA	CATGGC
'I 48h 3.1'	Total-RNA	ANOE-11	Small-RNA	CATTTT
'I 48h 5.1'	Total-RNA	ANOE-12	Small-RNA	CCAACA

Table 1: Description of the samples

** index sequences used for the basecalling*

3 Sequencing

3.1 Instrument

Serial number	**A00902**
Manufacturer	Illumina
Version	NovaSeq 6000
Slot used	A
Basecalling pipeline	- NovaSeq Control Software 1.7.0<br>- RTA v3.4.4<br>- bcl2fastq2.20 v2.20.0.422

3.2 Run

Run ID	**210511_A00902_A**
Mode	Standard
Number of cycles	2x50+8+8
Number of lanes	2
Flow cell ID	HCVL7DRXY
Flow cell version	S1
Kit version	NV3314690-RGSBS

3.3 Fasteris specifications[1]

Service	**Full flow-cell**
PF clusters (minimal yield)	**1'300 Mreads**
Q30	90 %

[1] Fasteris specifications for the run described in points above

4 Basecalling summary

4.1 Parameters

Lane	Number of mismatch in the index selection[1]	Phix spiked[2]	Other multiplexed libraries[3]
1	Dual 1	Yes	Yes
2	Dual 1	Yes	Yes

Table 2: Basecalling parameters
[1] According to Fasteris specifications, 1 mismatch is only authorized when all the indexes differ by at least 3 bases.
[2] Indicates if a PhiX reference is spiked (i.e. a low concentration of a PhiX library is added before sequencing and selectively retrieved through its related index) in your lane to estimate the error rate for your sequences.
[3] Indicates whether libraries from other projects are present in the lane

The base calling pipeline proceeds to the demultiplexing prior to the generation of fast-q sequence files, *i.e.* by separating the libraries according to their indexes. When different libraries are multiplexed in the same lane, a very low proportion of cross-talk may happen (reads sorted to wrong index)

4.2 Results

The sequences are sorted according to their index code.

Lane	Expected Read nb[1] (PF[2])	Library ID	Yield (Mb)	%PF	Cluster (PF)	Q30[3]	Mean qual. (PF)
1-2	18’000’000	ANOE-1	2'703	100	27'040'614	96.53	36.43
1-2	18’000’000	ANOE-2	2'341	100	23'417'461	96.50	36.42
1-2	18’000’000	ANOE-3	2'520	100	25'208'346	96.44	36.41
1-2	18’000’000	ANOE-4	2'313	100	23'141'894	96.65	36.45
1-2	18’000’000	ANOE-5	3'215	100	32'163'944	96.53	36.43
1-2	18’000’000	ANOE-6	2'837	100	28'385'563	95.99	36.33
1-2	18’000’000	ANOE-7	3'376	100	33'768'234	96.50	36.43
1-2	18’000’000	ANOE-8	3'027	100	30'284'517	96.21	36.37
1-2	18’000’000	ANOE-9	3'519	100	35'195'923	96.54	36.42
1-2	18’000’000	ANOE-10	2'484	100	24'854'623	96.52	36.43
1-2	18’000’000	ANOE-11	2'741	100	27'428'064	96.42	36.42
1-2	18’000’000	ANOE-12	2'645	100	26'462'268	96.28	36.39

[1] *Total number for all lanes combined*
[2] *PF stands for 'passed filter' i.e. clusters that fulfill the default Illumina quality criteria*
[3] *% of bases (PF) with a quality score greater or equal to 30*

4.3 Quality control

Spiked-PhiX:
Fasteris developed an "in-lane" control spike in each lane of the flow-cell. These spiked control reads are mapped on the PhiX reference genome. (Details can be found in the document "Fasteris_data-quality-and-specifications.pdf").

Q30:
The illumina pipelines estimates the reads quality according to the percentage of bases having a base quality value greater or equal to 30 (Q30), ie less than 1 error in 1000 bases. (c.f.: "Fasteris_data-quality-and-specifications.pdf").

Lane	Q30 Average %	Within specifications
1	95.08	Yes
2	94.46	Yes

N.B.: We do not perform quality filters on sequence files that are within our quality specifications, besides the default 'failed chastity' filter done by the pipeline itself. We have observed that additional filters can alter the representation/variability of the sequences (e.g. specific removal of sequences with secondary structures).

The base-calling pipeline can sometimes call bases as blanks ('N'). Blanks correspond to unattributed bases. If the blank rate is lower than 0.05%, no further information is provided.

5 Adapter trimming and inserts retrieval

Method
Our small RNA treatment uses the trimmomatic package to remove bases that correspond to the standard illumina adapters.

Trimmomatic	
Description	Trimmomatic: A flexible read trimming tool for Illumina NGS data
Version	0.32
Download Link	http://www.usadellab.org/cms/index.php?page=trimmomatic
Citing	Bolger, A. M., Lohse, M., & Usadel, B. (2014). Trimmomatic: A flexible trimmer for Illumina Sequence Data. *Bioinformatics*, btu170.

Trimmomatic will look for seed matches (<16 bases) allowing a defined number of mismatches.
In case of single end reads, whenever the seed alignment occurs, the entire alignment is scored. The trimming options used are: seedMismatches is set at 2 and simpleClipThreshold is at 5 (see Trimmomatic reference).

The inserts are sorted in separate sequence files according to their size. All inserts files from a library are grouped in a single compressed file.

The following table depicts the size distribution of inserts in bp (size window of 18 to 26 bases is usually of interest as it corresponds to miRNAs):

Insert range		0	1-17	18-26	27-50
L1-2_ANOE-10	# reads	52'131	1'237'812	16'946'057	6'618'623
	% reads	0.21	4.98	68.18	26.63
L1-2_ANOE-11	# reads	73'524	2'575'529	17'466'400	7'312'611
	% reads	0.27	9.39	63.68	26.66
L1-2_ANOE-12	# reads	89'485	2'900'999	18'374'609	5'097'175
	% reads	0.34	10.96	69.44	19.26
L1-2_ANOE-1	# reads	115'949	3'955'001	18'483'883	4'485'781
	% reads	0.43	14.63	68.36	16.59
L1-2_ANOE-2	# reads	73'641	4'610'793	14'669'615	4'063'412
	% reads	0.31	19.69	62.64	17.35
L1-2_ANOE-3	# reads	137'693	4'682'514	15'348'587	5'039'552
	% reads	0.55	18.58	60.89	19.99
L1-2_ANOE-4	# reads	91'786	1'678'013	15'775'441	5'596'654
	% reads	0.40	7.25	68.17	24.18
L1-2_ANOE-5	# reads	189'783	4'486'385	19'458'839	8'028'937
	% reads	0.59	13.95	60.50	24.96
L1-2_ANOE-6	# reads	213'640	7'210'729	16'793'082	4'168'112
	% reads	0.75	25.40	59.16	14.68
L1-2_ANOE-7	# reads	184'927	5'193'262	23'152'535	5'237'510
	% reads	0.55	15.38	68.56	15.51
L1-2_ANOE-8	# reads	136'326	5'097'097	19'093'052	5'958'042
	% reads	0.45	16.83	63.05	19.67
L1-2_ANOE-9	# reads	314'987	5'153'260	24'720'620	5'007'056
	% reads	0.89	14.64	70.24	14.23

A graphical representation of the inserts for your libraries is displayed below:

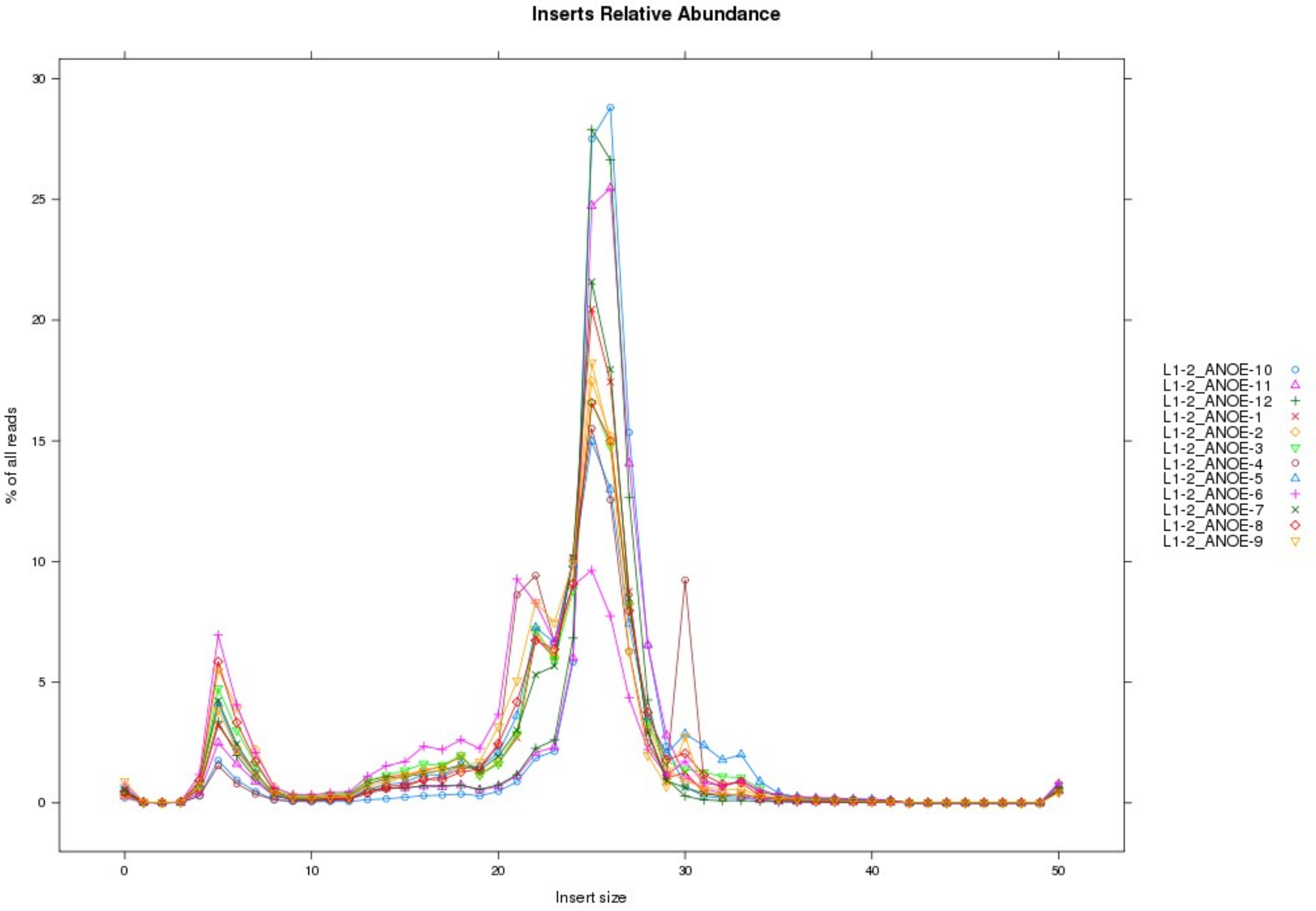

We observe one main peak at 25 bases. Shorter inserts are found around 16 bp and do most likely correspond to degradation products.

A histogram representation of the insert distribution per library is displayed below. The size of inserts is selected between 18 and 26 bases.

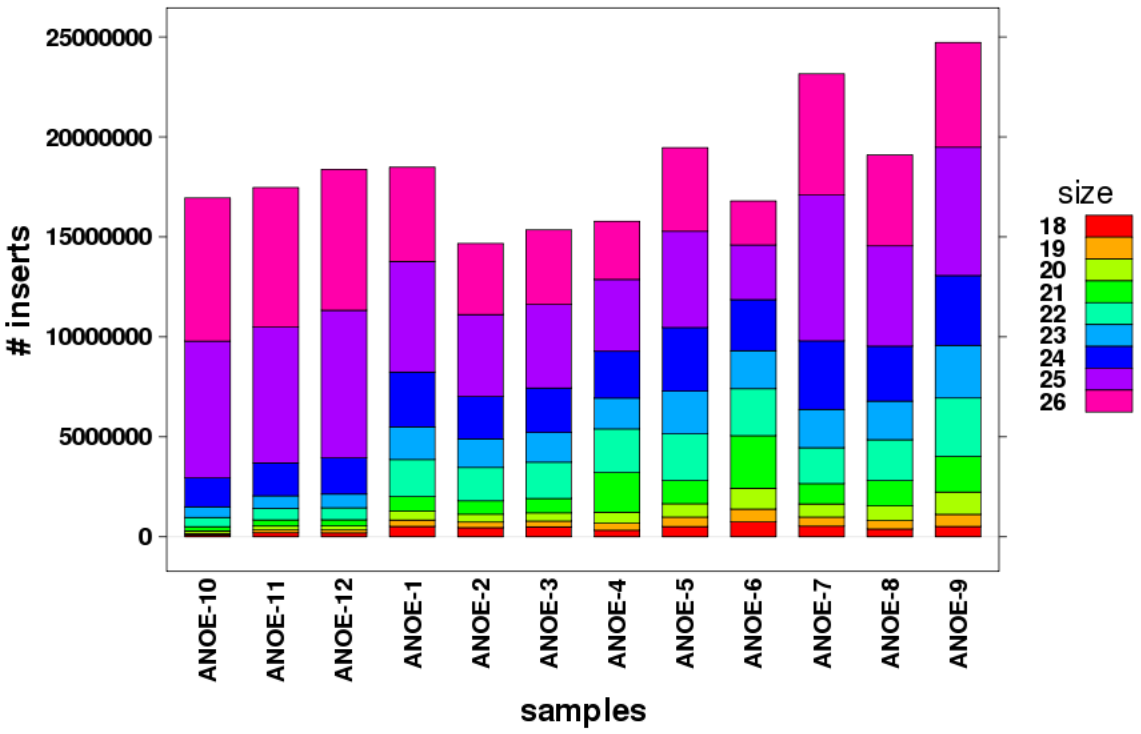

A similar histogram representation with the insert percentage per library is displayed below.

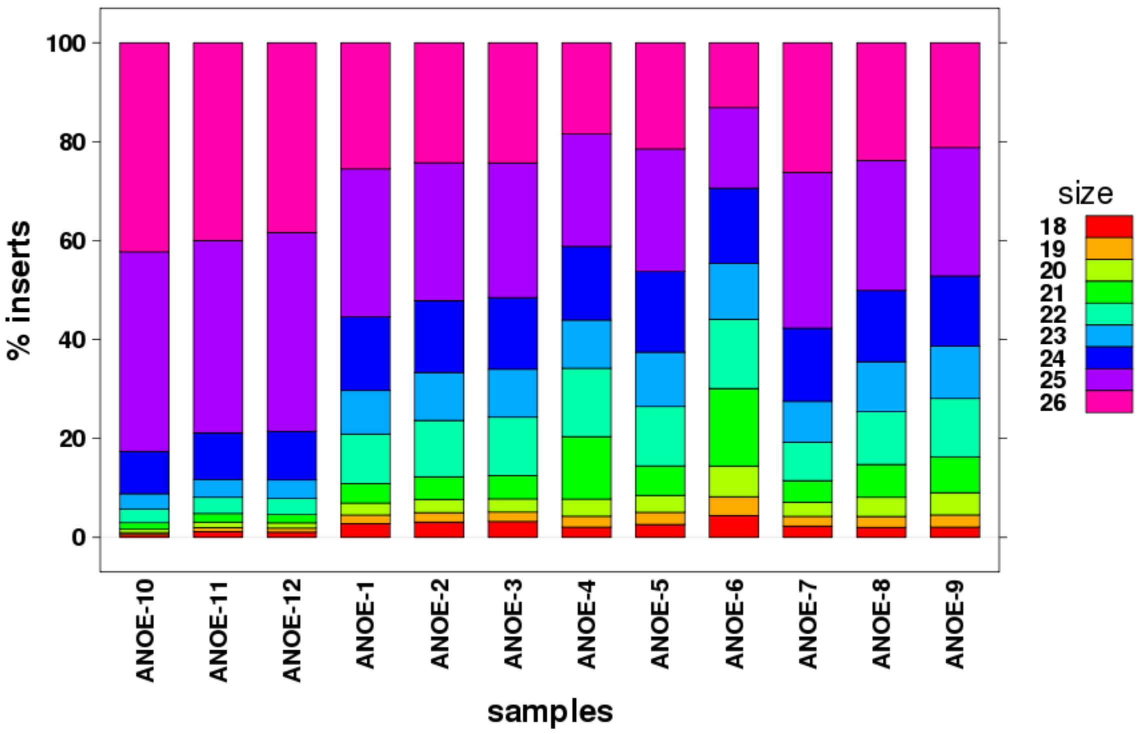

6 Nomenclature

Library:	The DNA or RNA samples are processed into short fragments of 20-1000 bp, depending on the protocols (e.g. genomic shotgun, transcriptome, ChIP-SEQ, small RNA, etc..) and cloned *in vitro* between the 3' and 5' adapters. Most libraries are amplified by PCR to generate enough DNA for precise measurement of its concentration, a key factor for maximizing the yield (too low concentration will mean not enough DNA clusters and too high will results in too many overlapping DNA clusters that will be eliminated at quality filter step during base-calling)
Insert:	Sample fragment that has been incorporated between two adapters during library preparation after fragmentation and size selection.
Adapter:	3' and 5' sequences added during library preparation (used for PCR amplification, DNA cluster generation on the flow cell and sequencing)
Index:	A 6-to-8 bases DNA sequence tag found in adapter sequence to uniquely identify each library. The index is read separately from the inserts and its sequence is used at demultiplexing step.
DNA Cluster:	A DNA Colony generated on the flow-cell from a single DNA molecule of the library. It is perceived as a single sequencing unit by the base-calling pipeline, even if 2 DNA clusters are overlapping. In such case, the double sequence produced is eliminated at filtering step.
Read:	A sequence obtained after base calling. Its length is determined by the number of sequencing cycles. All the reads have the same length.<br>Runs done using only forward sequencing primer, will generate one **single-read** per DNA cluster. Runs done using the forward and the reverse sequencing primers will produce two **paired-reads** (one pair) per DNA cluster.
PF Clusters:	Pass filter clusters. The illumina pipeline uses the chastity filter *(c.f.: "FAS_MKT_X_NGS-QCandSpecifications.pdf")* to remove sequences produced from clusters with low signal to noise ratio (e.g. overlapping DNA clusters).
PhiX Spike:	A Fasteris-developped quality control to measure the real error rate in the lane. About 0.5% of a PhiX library is added in each lane.
Demultiplexing:	Sorting the reads according to the indexes of each library

7 File format

**Index selected sequences**<br>e.g. 210511_A00902_A_L1-2_ANOE-1_R1.fastq.gz<br>**Inserts (i.e. reads where the adapter was found and removed)**<br>e.g. 210511_A00902_A_L1-2_ANOE-1_R1.InsertSize17.fastq<br>**In a single compressed file per library**<br>e.g. 2021-05-17_L1-2_ANOE-1_inserts.tar.gz
File extension: fastq.gz
Format: Illumina fast-q format. A text file listing the sequences and their quality. Each group of 4 lines describe one sequence:<br>*Line 1: is the sequence name (a unique identifier of the sequence)*<br>*Line 2: the base sequence itself*<br>*Line 3: orientation of the sequence (always + to indicate forward strand)*<br>*Line 4: quality value for each base, encoded as a Phred score*

The sequence files are available to download on our secured server.

N.B. The sequence files are compressed as .gz archives. The archives can be uncompressed on linux OS using a gzip -d command. We cannot guarantee, due to their large size, that they can be uncompressed on Windows or MacOS systems.
