# Supplementary material for: The intracellular parasite Anncaliia algerae induces a massive miRNA down-regulation in human cells
Source: Noncoding RNA Res. 2023 May 18;8(3):363–75. doi: 10.1016/j.ncrna.2023.05.003 (PMC10238475; doi:10.1016/j.ncrna.2023.05.003)
Supplement: Multimedia component 5 [file mmc5.zip › S5 File/SmallRNAseq/DR_2021-03-10_ANOE-1-19+21-24+49.pdf]

Illumina Services<br>**Data Report**
ANOE-20210210a

Customer		Fasteris	
Account	Eric Peyretaillade	Project	ANOE-20210210a
		Date / ref.	DR_2021-03-10_ANOE-1-19+21-24+49
		Author	Thibault Robin

Summary

Sequenced libraries	**ANOE-1-19+21-24+49**
Number of libraries	**24**
Sample type	**Total-RNA**
Run ID	**210305_A00902_B**
Number of cycle	**2x100+8+8**
Instrument	**NovaSeq**
Yield(GB)	**65.8**
Quality control	**Within specifications**

Author's comments:

No comment

1 Introduction

DNA or RNA samples are processed using illumina technology to prepare libraries, which are then sequenced on Next-Generation DNA sequencing (NGS) instruments.

The present report describes the pipeline used and provides quality and yield information about the data produced.

Upon request, our bioinformatics team is happy to help you to extract the biologically useful information from your data.

NB: Although the illumina NGS instruments deliver high-quality data, you must obtain independent validation of the data.

Illumina Propel Certified™ 2017 MiSeq® NextSeq® HiSeq® 2500 HiSeq® 4000

2 Samples

Sample name	Sample type	Library ID	Library type	Index *
'I 3h 1.1'	Total-RNA	ANOE-1	Small-RNA	ACTGATATGGGAAAGT
'I 3h 3.1'	Total-RNA	ANOE-2	Small-RNA	ATGAGCATGGGAAAGT
'I 3h 5.1'	Total-RNA	ANOE-3	Small-RNA	ATTCCTATGGGAAAGT
'I 12h 1.1'	Total-RNA	ANOE-4	Small-RNA	CAAAAGATGGGAAAGT
'I 12h 3.1'	Total-RNA	ANOE-5	Small-RNA	CAACTAATGGGAAAGT
'I 12h 5.1'	Total-RNA	ANOE-6	Small-RNA	CACCGGATGGGAAAGT
'I 24h 1.1'	Total-RNA	ANOE-7	Small-RNA	CACGATATGGGAAAGT
'I 24h 3.1'	Total-RNA	ANOE-8	Small-RNA	CACTCAATGGGAAAGT
'I 24h 4.1'	Total-RNA	ANOE-9	Small-RNA	CAGGCGATGGGAAAGT
'I 48h 2.1'	Total-RNA	ANOE-10	Small-RNA	CATGGCATGGGAAAGT
'I 48h 3.1'	Total-RNA	ANOE-11	Small-RNA	CATTTTATGGGAAAGT
'I 48h 5.1'	Total-RNA	ANOE-12	Small-RNA	CCAACAATGGGAAAGT
'3h NI 3.1'	Total-RNA	ANOE-13	Small-RNA	CGGAATATGGGAAAGT
'3h NI 4.1'	Total-RNA	ANOE-14	Small-RNA	CTAGCTATGGGAAAGT
'3h NI 5.1'	Total-RNA	ANOE-15	Small-RNA	CTATACATGGGAAAGT
'12h NI 1.1'	Total-RNA	ANOE-16	Small-RNA	CTCAGAATGGGAAAGT
'12h NI 2.1'	Total-RNA	ANOE-17	Small-RNA	GACGACATGGGAAAGT
'12h NI 3.1'	Total-RNA	ANOE-18	Small-RNA	TAATCGATGGGAAAGT
'24h NI 1.1'	Total-RNA	ANOE-19	Small-RNA	TACAGCATGGGAAAGT
'24h NI 5.1'	Total-RNA	ANOE-21	Small-RNA	TATAATATGGGAAAGT
'48h NI 1.1'	Total-RNA	ANOE-22	Small-RNA	TCATTCATGGGAAAGT
'48h NI 2.1'	Total-RNA	ANOE-23	Small-RNA	TCCCGAATGGGAAAGT
'48h NI 3.1'	Total-RNA	ANOE-24	Small-RNA	TCGAAGATGGGAAAGT
'ANOE-20_24h NI 2.1_2ndbatch'	Total-RNA	ANOE-49	Small-RNA	TCGGCAATGGGAAAGT

Table 1: Description of the samples

** index sequences used for the basecalling*

3 Sequencing specifications

3.1 Instrument

Serial number	**A00902**
Manufacturer	Illumina
Version	NovaSeq 6000
Slot used	B
Basecalling pipeline	- NovaSeq Control Software 1.7.0<br>- RTA v3.4.4<br>- bcl2fastq2.20 v2.20.0.422

3.2 Run

Run ID	**210305_A00902_B**
Mode	Xp
Number of cycles	2x100+8+8
Number of lanes	1
Flow cell ID	H3KHWDRXY
Flow cell version	SP
Kit version	NV3171844-RGSBS

3.3 Fasteris specifications[1]

Service	**Full flow-cell**
PF clusters (minimal yield)	650 Mreads
Q30	80 %

[1] Fasteris specifications for the run described in points above

4 Basecalling summary

4.1 Parameters

Lane	Number of mismatch in the index selection[1]	Phix spiked[2]	Other multiplexed libraries[3]
1	0	Yes	Yes

Table 2: Basecalling parameters

[1] *According to Fasteris specifications, 1 mismatch is only authorized when all the indexes differ by at least 3 bases.*
[2] *Indicates if a PhiX reference is spiked (i.e. a low concentration of a PhiX library is added before sequencing and selectively retrieved through its related index) in your lane to estimate the error rate for your sequences.*
[3] *Indicates whether libraries from other projects are present in the lane*

The base calling pipeline proceeds to the demultiplexing prior to the generation of fast-q sequence files, *i.e.* by separating the libraries according to their indexes. When different libraries are multiplexed in the same lane, a very low proportion of cross-talk may happen (reads sorted to wrong index)

4.2 Results

The sequences are sorted according to their index code.

Lane	Expected Read nb[1] (PF[2])	Library ID	Yield (Mb)	%PF	Cluster (PF)	Q30[3]	Mean qual. (PF)
1	8’000’000	ANOE-1	2'427	100	12'136'677	89.39	35.07
1	8’000’000	ANOE-2	1'749	100	8'746'304	88.70	34.93
1	8’000’000	ANOE-3	2'298	100	11'493'495	88.95	34.98
1	8’000’000	ANOE-4	2'565	100	12'825'084	89.31	35.05
1	8’000’000	ANOE-5	3'140	100	15'701'699	87.04	34.61
1	8’000’000	ANOE-6	2'625	100	13'125'058	83.01	33.70
1	8’000’000	ANOE-7	3'972	100	19'863'277	89.36	35.05
1	8’000’000	ANOE-8	3'057	100	15'285'874	85.65	34.31
1	8’000’000	ANOE-9	3'019	100	15'098'052	86.99	34.60
1	8’000’000	ANOE-10	2'473	100	12'368'221	88.58	34.93
1	8’000’000	ANOE-11	2'582	100	12'910'974	89.56	35.11
1	8’000’000	ANOE-12	2'650	100	13'250'792	86.88	34.59
1	8’000’000	ANOE-13	3'071	100	15'356'040	88.28	34.81
1	8’000’000	ANOE-14	2'766	100	13'830'371	87.99	34.77
1	8’000’000	ANOE-15	2'873	100	14'368'253	88.28	34.81
1	8’000’000	ANOE-16	2'177	100	10'889'999	79.61	32.75
1	8’000’000	ANOE-17	2'581	100	12'905'688	88.91	34.95
1	8’000’000	ANOE-18	2'367	100	11'839'769	86.87	34.57
1	8’000’000	ANOE-19	2'872	100	14'361'579	85.51	34.28
1	8’000’000	ANOE-21	3'313	100	16'569'366	88.41	34.85
1	8’000’000	ANOE-22	3'020	100	15'104'506	85.36	34.25
1	8’000’000	ANOE-23	2'607	100	13'038'728	80.23	32.90
1	8’000’000	ANOE-24	2'899	100	14'499'472	88.26	34.82
1	8’000’000	ANOE-49	2'684	100	13'421'338	85.06	34.19

[1] *The expected read number corresponds to the total number for all lanes combined*
[2] *PF stands for 'passed filter' i.e. clusters that fulfill the default Illumina quality criteria*
[3] *% of bases (PF) with a quality score greater or equal to 30*

4.3 Quality control

Spiked-PhiX:
Fasteris developed an "in-lane" control spike in each lane of the flow-cell. These spiked control reads are mapped on the PhiX reference genome. (Details can be found in the document "Fasteris_data-quality-and-specifications.pdf").

Q30:
The illumina pipelines estimates the reads quality according to the percentage of bases having a base quality value greater or equal to 30 (Q30), ie less than 1 error in 1000 bases. (c.f. : "Fasteris_data-quality-and-specifications.pdf").

Lane	Q30 Average %	Within specifications
1	89.46	Yes

N.B.: We do not perform quality filters on sequence files that are within our quality specifications, besides the default 'failed chastity' filter done by the pipeline itself. We have observed that additional filters can alter the representation/variability of the sequences (e.g. specific removal of sequences with secondary structures).

The base-calling pipeline can sometimes call bases as blanks ('N'). Blanks correspond to unattributed bases. If the blank rate is lower than 0.05%, no further information is provided.

5 Adapter trimming and Inserts retrieval

Method
Our small RNA treatment uses the trimmomatic package to remove bases that correspond to the standard illumina adapters.

Trimmomatic	
Description	Trimmomatic: A flexible read trimming tool for Illumina NGS data
Version	0.32
Download Link	http://www.usadellab.org/cms/index.php?page=trimmomatic
Citing	Bolger, A. M., Lohse, M., & Usadel, B. (2014). Trimmomatic: A flexible trimmer for Illumina Sequence Data. *Bioinformatics*, btu170.

Trimmomatic will look for seed matches (<16 bases) allowing a defined number of mismatches.
In case of single end reads, whenever the seed alignment occurs, the entire alignment is scored. The trimming options used are: seedMismatches is set at 2 and simpleClipThreshold is at 5 (see Trimmomatic reference).

The inserts are sorted in separate sequence files according to their size. All inserts files from a library are grouped in a single compressed file.

The following table depicts the size distribution of inserts in bp (size window of 18 to 26 bases is usually of interest as it corresponds to miRNAs) :

Insert range		0	1-17	18-26	27-50
L001_ANOE-10	# reads	30'524	639'341	8'398'616	3'299'740
	% reads	0.25	5.17	67.90	26.68
L001_ANOE-11	# reads	38'826	1'254'191	8'170'503	3'447'454
	% reads	0.30	9.71	63.28	26.70
L001_ANOE-12	# reads	48'769	1'445'407	9'168'925	2'587'691
	% reads	0.37	10.91	69.20	19.53
L001_ANOE-13	# reads	286'890	7'503'324	6'387'495	1'178'331
	% reads	1.87	48.86	41.60	7.67
L001_ANOE-14	# reads	199'010	5'380'440	6'340'136	1'910'785
	% reads	1.44	38.90	45.84	13.82
L001_ANOE-15	# reads	126'129	5'586'022	7'703'175	952'927
	% reads	0.88	38.88	53.61	6.63
L001_ANOE-16	# reads	187'246	4'877'346	4'273'407	1'552'000
	% reads	1.72	44.79	39.24	14.25
L001_ANOE-17	# reads	176'621	3'997'009	4'921'460	3'810'598
	% reads	1.37	30.97	38.13	29.53
L001_ANOE-18	# reads	222'704	3'918'498	6'073'041	1'625'526
	% reads	1.88	33.10	51.29	13.73
L001_ANOE-19	# reads	155'045	5'248'283	7'169'626	1'788'625
	% reads	1.08	36.54	49.92	12.45
L001_ANOE-1	# reads	54'373	1'827'437	8'236'339	2'018'528
	% reads	0.45	15.06	67.86	16.63
L001_ANOE-21	# reads	190'413	5'238'752	7'442'019	3'698'182
	% reads	1.15	31.62	44.91	22.32
L001_ANOE-22	# reads	11'642'499	1'118'900	1'761'523	581'584
	% reads	77.08	7.41	11.66	3.85
L001_ANOE-23	# reads	136'776	5'225'853	6'117'522	1'558'577
	% reads	1.05	40.08	46.92	11.95
L001_ANOE-24	# reads	201'413	5'903'591	5'408'932	2'985'536
	% reads	1.39	40.72	37.30	20.59
L001_ANOE-2	# reads	30'981	1'774'650	5'423'116	1'517'557

Insert range		0	1-17	18-26	27-50
	% reads	0.35	20.29	62.00	17.35
L001_ANOE-3	# reads	65'145	2'189'176	6'932'279	2'306'895
	% reads	0.57	19.05	60.31	20.07
L001_ANOE-49	# reads	113'163	5'713'093	6'623'303	971'779
	% reads	0.84	42.57	49.35	7.24
L001_ANOE-4	# reads	54'672	963'666	8'679'275	3'127'471
	% reads	0.43	7.51	67.67	24.39
L001_ANOE-5	# reads	96'947	2'235'211	9'424'029	3'945'512
	% reads	0.62	14.24	60.02	25.13
L001_ANOE-6	# reads	103'924	3'392'076	7'687'663	1'941'395
	% reads	0.79	25.84	58.57	14.79
L001_ANOE-7	# reads	115'133	3'137'234	13'511'425	3'099'485
	% reads	0.58	15.79	68.02	15.60
L001_ANOE-8	# reads	73'994	2'607'711	9'563'203	3'040'966
	% reads	0.48	17.06	62.56	19.89
L001_ANOE-9	# reads	140'079	2'297'326	10'497'265	2'163'382
	% reads	0.93	15.22	69.53	14.33

A graphical representation of the inserts for your libraries is displayed below:

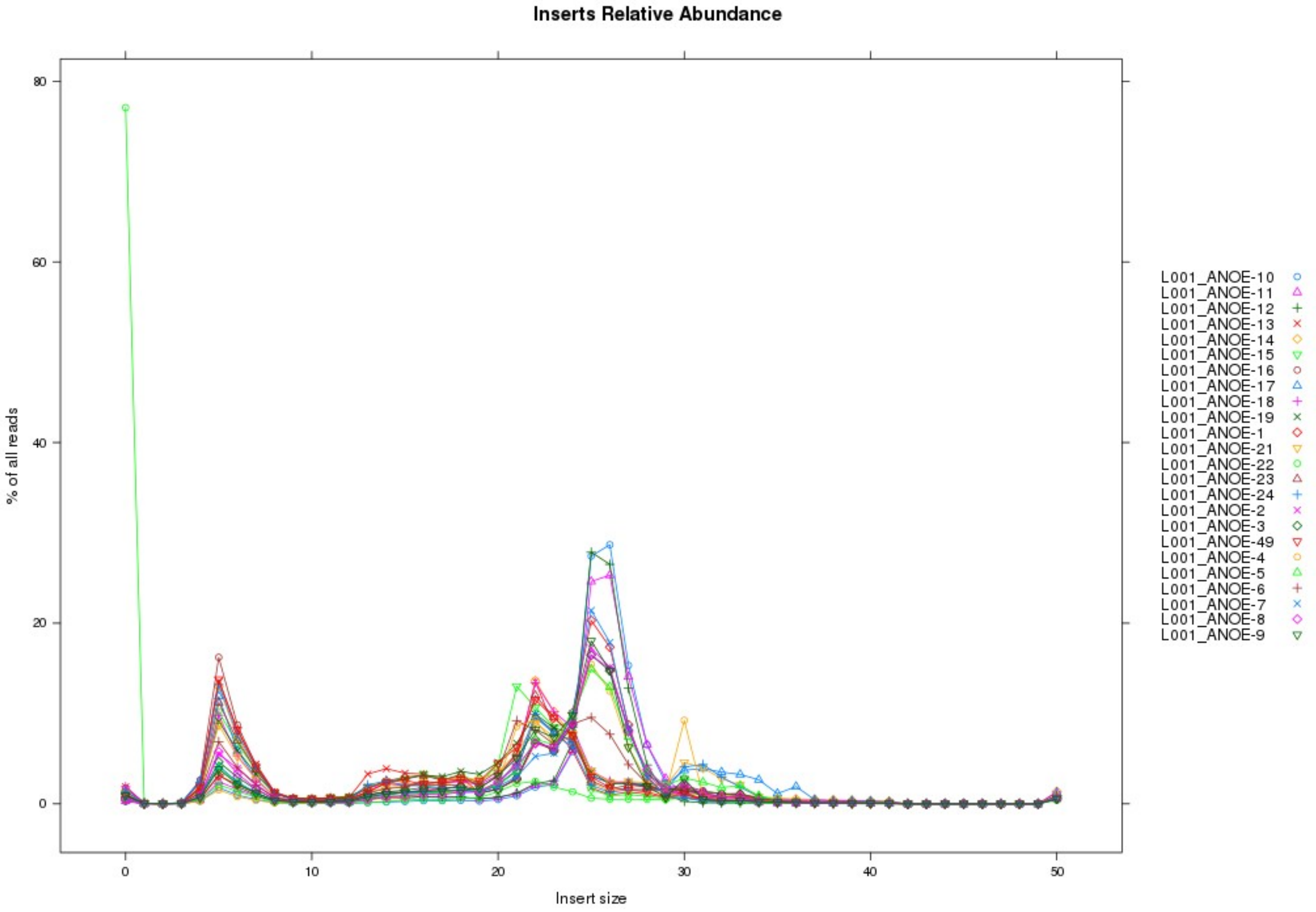

We observe two peaks, respectively at 22 and 27 bases. Shorter inserts are found below 20bp and do most likely correspond to degradation products.

A histogram representation of the insert distribution per library is displayed below. The size of inserts is selected between 18 and 26 bases.

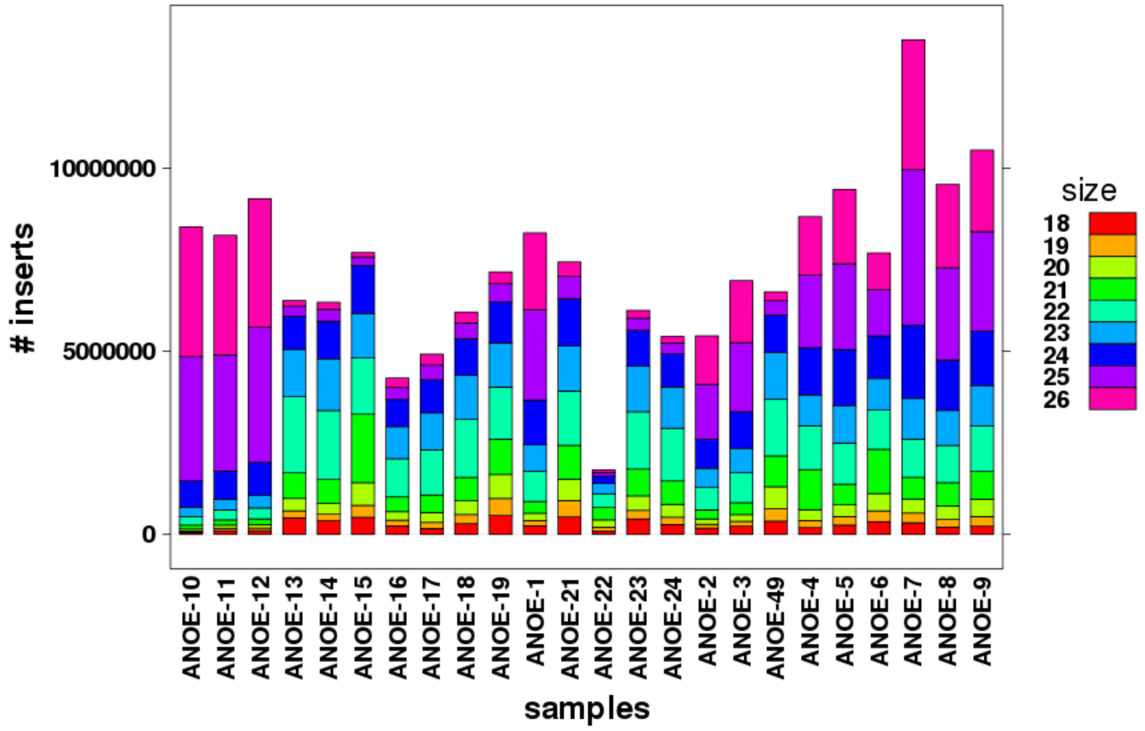

A similar histogram representation with the insert percentage per library is displayed below.

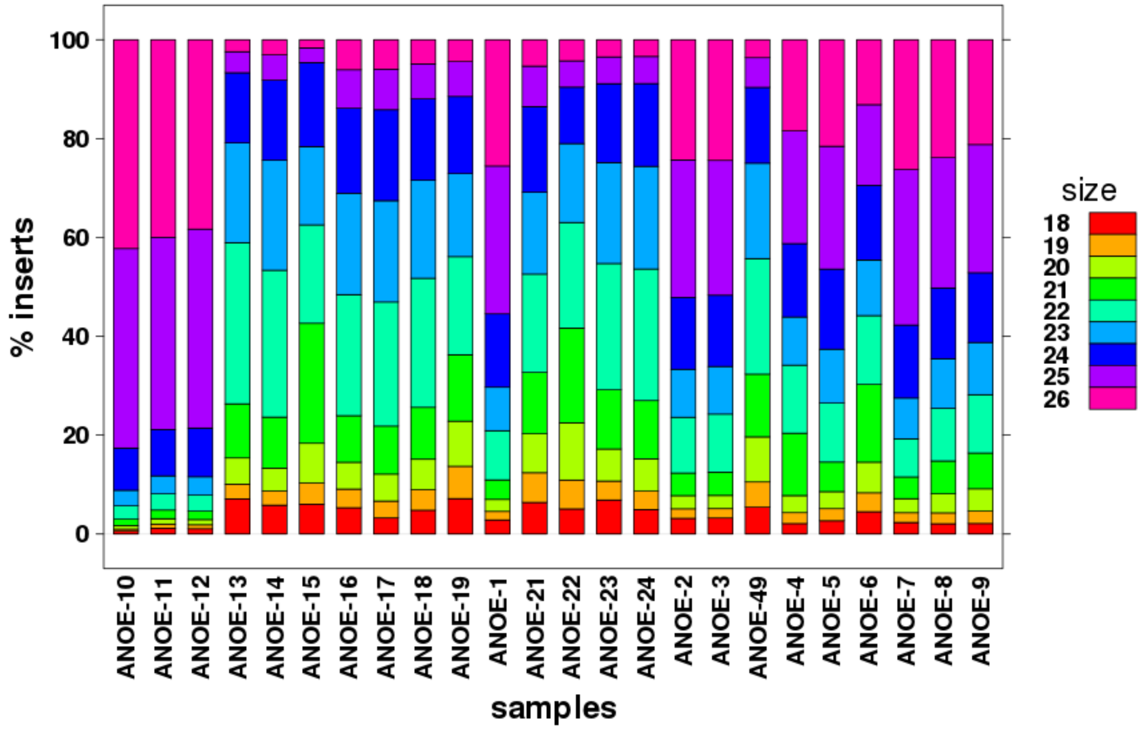

6 Nomenclature

Library:	The DNA or RNA samples are processed into short fragments of 20-1000 bp, depending on the protocols (e.g. genomic shotgun, transcriptome, ChIP-SEQ, small RNA, etc..) and cloned *in vitro* between the 3' and 5' adapters. Most libraries are amplified by PCR to generate enough DNA for precise measurement of its concentration, a key factor for maximizing the yield (too low concentration will mean not enough DNA clusters and too high will results in too many overlapping DNA clusters that will be eliminated at quality filter step during base-calling)
Insert:	Sample fragment that has been incorporated between two adapters during library preparation after fragmentation and size selection.
Adapter:	3' and 5' sequences added during library preparation (used for PCR amplification, DNA cluster generation on the flow cell and sequencing)
Index:	A 6-to-8 bases DNA sequence tag found in adapter sequence to uniquely identify each library. The index is read separately from the inserts and its sequence is used at demultiplexing step.
DNA Cluster:	A DNA Colony generated on the flow-cell from a single DNA molecule of the library. It is perceived as a single sequencing unit by the base-calling pipeline, even if 2 DNA clusters are overlapping. In such case, the double sequence produced is eliminated at filtering step.
Read:	A sequence obtained after base calling. Its length is determined by the number of sequencing cycles. All the reads have the same length.<br>Runs done using only forward sequencing primer, will generate one **single-read** per DNA cluster. Runs done using the forward and the reverse sequencing primers will produce two **paired-reads** (one pair) per DNA cluster.
PF Clusters:	Pass filter clusters. The illumina pipeline uses the chastity filter *(c.f. : “FAS_MKT_X_NGS-QCandSpecifications.pdf”)* to remove sequences produced from clusters with low signal to noise ratio (e.g. overlapping DNA clusters).
PhiX Spike:	A Fasteris-developped quality control to measure the real error rate in the lane. About 0.5% of a PhiX library is added in each lane.
Demultiplexing:	Sorting the reads according to the indexes of each library

7 File format

**Index selected sequences**<br>e.g. 210305_A00902_B_L001_ANOE-1_R1.fastq.gz<br>**Inserts (i.e. Reads where the adapter was found and removed)**<br>e.g. 210305_A00902_B_L001_ANOE-1_R1.InsertSize17.fastq<br>**In a single compressed file per library**<br>e.g. 2021-03-09_L001_ANOE-1_inserts.tar.gz
File extension: fastq.gz
Format: Illumina fast-q format. A text file listing the sequences and their quality. Each group of 4 lines describe one sequence:<br>*Line 1: is the sequence name (a unique identifier of the sequence)*<br>*Line 2: the base sequence itself*<br>*Line 3- orientation of the sequence (always + to indicate forward strand)*<br>*Line 4- quality value for each base, encoded as a Phred score*

The sequence files are available to download on our secured server.

N.B. The sequence files are compressed as .gz archives. The archives can be uncompressed on linux OS using a gzip -d command. We cannot guarantee, due to their large size, that they can be uncompressed on Windows or MacOS systems.
