# Supplementary material for: The intracellular parasite Anncaliia algerae induces a massive miRNA down-regulation in human cells
Source: Noncoding RNA Res. 2023 May 18;8(3):363–75. doi: 10.1016/j.ncrna.2023.05.003 (PMC10238475; doi:10.1016/j.ncrna.2023.05.003)
Supplement: Multimedia component 5 [file mmc5.zip › S5 File/PolyASeq/DR_2021-05-18_ANOE-28-36 (1).pdf]

Illumina Services<br>**Data Report**
ANOE-20210210b

Customer		Fasteris	
Account	Eric Peyretaillade	Project	ANOE-20210210b
		Date / ref.	DR_2021-05-18_ANOE-28-36
		Author	Thibault Robin

Summary

Sequenced libraries	**ANOE-28-36**
Number of libraries	**9**
Sample type	**Total-RNA**
Run ID	**210511_A00902_A**
Number of cycle	**2x50+8+8**
Instrument	**NovaSeq**
Yield(GB)	**153.2**
Quality control	**Within specifications**

Author's comments:

No comment

1 Introduction

DNA or RNA samples are processed using illumina technology to prepare libraries, which are then sequenced on Next-Generation DNA sequencing (NGS) instruments.

The present report describes the pipeline used and provides quality and yield information about the data produced.

Upon request, our bioinformatics team is happy to help you to extract the biologically useful information from your data.

NB: Although the illumina NGS instruments deliver high-quality data, you must obtain independent validation of the data.

2 Library preparation

Sample name	Sample type	Library ID	Library type	Index *
'I 12h 1.2'	Total-RNA	ANOE-28	Stranded_mRNA	AAGTCCAATACTCATA
'I 12h 3.2'	Total-RNA	ANOE-29	Stranded_mRNA	ATCCACTGACGCACCT
'I 12h 5.2'	Total-RNA	ANOE-30	Stranded_mRNA	GCTTGTCAGTATGTTC
'I 24h 1.2'	Total-RNA	ANOE-31	Stranded_mRNA	CAAGCTAGCGCTATGT
'I 24h 3.2'	Total-RNA	ANOE-32	Stranded_mRNA	TGGATCGATATCGCAC
'I 24h 4.2'	Total-RNA	ANOE-33	Stranded_mRNA	AGTTCAGGTCTGTTGG
'I 48h 2.2'	Total-RNA	ANOE-34	Stranded_mRNA	GACCTGAACTCACCAA
'I 48h 3.2'	Total-RNA	ANOE-35	Stranded_mRNA	TCTCTACTGAACCGCG
'I 48h 5.2'	Total-RNA	ANOE-36	Stranded_mRNA	CTCTCGTCAGGTTATA

Table 1: Description of the samples

** index sequences used for the basecalling*

3 Sequencing

3.1 Instrument

Serial number	**A00902**
Manufacturer	Illumina
Version	NovaSeq 6000
Slot used	A
Basecalling pipeline	- NovaSeq Control Software 1.7.0<br>- RTA v3.4.4<br>- bcl2fastq2.20 v2.20.0.422

3.2 Run

Run ID	**210511_A00902_A**
Mode	Standard
Number of cycles	2x50+8+8
Number of lanes	2
Flow cell ID	HCVL7DRXY
Flow cell version	S1
Kit version	NV3314690-RGSBS

3.3 Fasteris specifications[1]

Service	**Full flow-cell**
PF clusters (minimal yield)	**1'300 Mreads**
Q30	85 %

[1] Fasteris specifications for the run described in points above

4 Basecalling summary

4.1 Parameters

Lane	Number of mismatch in the index selection[1]	Phix spiked[2]	Other multiplexed libraries[3]
1	Dual 1	Yes	Yes
2	Dual 1	Yes	Yes

Table 2: Basecalling parameters
[1] According to Fasteris specifications, 1 mismatch is only authorized when all the indexes differ by at least 3 bases.
[2] Indicates if a PhiX reference is spiked (i.e. a low concentration of a PhiX library is added before sequencing and selectively retrieved through its related index) in your lane to estimate the error rate for your sequences.
[3] Indicates whether libraries from other projects are present in the lane

The base calling pipeline proceeds to the demultiplexing prior to the generation of fast-q sequence files, *i.e.* by separating the libraries according to their indexes. When different libraries are multiplexed in the same lane, a very low proportion of cross-talk may happen (reads sorted to wrong index)

4.2 Results

The sequences are sorted according to their index code.

Lane	Expected Read nb[1] (PF[2])	Library ID	Yield (Mb)	%PF	Cluster (PF)	Q30[3]	Mean qual. (PF)
1-2	217’000’000	ANOE-28	28'564	100	285'656'382	91.44	35.32
1-2	217’000’000	ANOE-29	27'728	100	277'295'445	91.90	35.44
1-2	217’000’000	ANOE-30	29'429	100	294'296'326	92.56	35.58
1-2	72’000’000	ANOE-31	10'618	100	106'193'027	92.88	35.65
1-2	72’000’000	ANOE-32	10'437	100	104'382'062	93.44	35.79
1-2	72’000’000	ANOE-33	10'437	100	104'376'145	92.79	35.65
1-2	72’000’000	ANOE-34	11'239	100	112'406'361	92.66	35.61
1-2	72’000’000	ANOE-35	12'925	100	129'264'457	92.43	35.57
1-2	72’000’000	ANOE-36	11'832	100	118'331'407	91.42	35.31

[1] Total number for all lanes combined
[2] PF stands for 'passed filter' i.e. clusters that fulfill the default Illumina quality criteria
[3] % of bases (PF) with a quality score greater or equal to 30

4.3 Quality control

Spiked-PhiX:
Fasteris developed an “in-lane” control spike in each lane of the flow-cell. These spiked control reads are mapped on the PhiX reference genome. (Details can be found in the document “Fasteris_data-quality-and-specifications.pdf”).

Q30:
The illumina pipelines estimates the reads quality according to the percentage of bases having a base quality value greater or equal to 30 (Q30), ie less than 1 error in 1000 bases. (c.f.: “Fasteris_data-quality-and-specifications.pdf”).

Lane	Q30 Average %	Within specifications
1	95.08	Yes
2	94.46	Yes

N.B.: We do not perform quality filters on sequence files that are within our quality specifications, besides the default 'failed chastity' filter done by the pipeline itself. We have observed that additional filters can alter the representation/variability of the sequences (e.g. specific removal of sequences with secondary structures).

The base-calling pipeline can sometimes call bases as blanks ('N'). Blanks correspond to unattributed bases. If the blank rate is lower than 0.05%, no further information is provided.

5 Nomenclature

Library:	The DNA or RNA samples are processed into short fragments of 20-1000 bp, depending on the protocols (e.g. genomic shotgun, transcriptome, ChIP-SEQ, small RNA, etc..) and cloned *in vitro* between the 3' and 5' adapters. Most libraries are amplified by PCR to generate enough DNA for precise measurement of its concentration, a key factor for maximizing the yield (too low concentration will mean not enough DNA clusters and too high will results in too many overlapping DNA clusters that will be eliminated at quality filter step during base-calling)
Insert:	Sample fragment that has been incorporated between two adapters during library preparation after fragmentation and size selection.
Adapter:	3' and 5' sequences added during library preparation (used for PCR amplification, DNA cluster generation on the flow cell and sequencing)
Index:	A 6-to-8 bases DNA sequence tag found in adapter sequence to uniquely identify each library. The index is read separately from the inserts and its sequence is used at demultiplexing step.
DNA Cluster:	A DNA Colony generated on the flow-cell from a single DNA molecule of the library. It is perceived as a single sequencing unit by the base-calling pipeline, even if 2 DNA clusters are overlapping. In such case, the double sequence produced is eliminated at filtering step.
Read:	A sequence obtained after base calling. Its length is determined by the number of sequencing cycles. All the reads have the same length.<br>Runs done using only forward sequencing primer, will generate one **single-read** per DNA cluster. Runs done using the forward and the reverse sequencing primers will produce two **paired-reads** (one pair) per DNA cluster.
PF Clusters:	Pass filter clusters. The illumina pipeline uses the chastity filter *(c.f.: "FAS_MKT_X_NGS-QCandSpecifications.pdf")* to remove sequences produced from clusters with low signal to noise ratio (e.g. overlapping DNA clusters).
PhiX Spike:	A Fasteris-developped quality control to measure the real error rate in the lane. About 0.5% of a PhiX library is added in each lane.
Demultiplexing:	Sorting the reads according to the indexes of each library

6 File format

**Index selected sequences**<br>e.g. 210511_A00902_A_L1-2_ANOE-28_R1.fastq.gz<br>210511_A00902_A_L1-2_ANOE-28_R2.fastq.gz
File extension: fastq.gz
Format: Illumina fast-q format. A text file listing the sequences and their quality. Each group of 4 lines describe one sequence:<br>*Line 1: is the sequence name (a unique identifier of the sequence)*<br>*Line 2: the base sequence itself*<br>*Line 3: orientation of the sequence (always + to indicate forward strand)*<br>*Line 4: quality value for each base, encoded as a Phred score*

The sequence files are available to download on our secured server.

N.B. The sequence files are compressed as .gz archives. The archives can be uncompressed on linux OS using a gzip -d command. We cannot guarantee, due to their large size, that they can be uncompressed on Windows or MacOS systems.
